# Internet severity and activities addiction questionnaire (ISAAQ): Psychometrics of item response theory and clustering of online activities

**DOI:** 10.1016/j.comppsych.2023.152366

**Published:** 2023-04

**Authors:** Konstantinos Ioannidis, Jeggan Tiego, Nina Lutz, Charlene Omrawo, Murat Yücel, Jon E. Grant, Christine Lochner, Samuel R. Chamberlain

**Affiliations:** a Southern Health NHS Foundation Trust, Southampton, UK; b Turner Institute for Brain and Mental Health and School of Psychological Sciences, Monash University, Clayton, VIC, Australia; c Department of Psychiatry, University of Cambridge, UK; d SA MRC Unit on Risk and Resilience in Mental Disorders, Department of Psychiatry, University of Stellenbosch, South Africa; e Department of Psychiatry and Behavioral Neuroscience, University of Chicago, Chicago, IL, USA; f Department of Psychiatry, Faculty of Medicine, University of Southampton, UK

**Keywords:** problematic use of the internet, internet addiction, item response theory, confirmatory factor analysis, reliability, validity

## Abstract

**Background:**

Problematic usage of the internet (PUI) is an umbrella term, referring to a variety of maladaptive online behaviors linked to functional impairment. There is ongoing need for the development of instruments capturing not only PUI severity, but also the online activity types. The Internet Severity and Activities Questionnaire (ISAAQ), previously developed to address this need, required further refinement and validation.

**Methods:**

Cross-sectional data was gathered in two separate samples (South Africa *n* = 3275, USA-UK *n* = 943) using the Internet Severity and Activities Addiction Questionnaire (ISAAQ). Item Response Theory (IRT) was used to examine the properties of the scale (Part A of the ISAAQ) and differential item functioning against demographic parameters. The severity scale of the ISAAQ was optimized by eliminating the poorest performing items using an iterative approach and examining validity metrics. Cluster analyses was used to examine internet activities and commonalities across samples (Part B of the ISAAQ).

**Results:**

Optimization of ISAAQ using IRT yielded a refined 10-item version (ISAAQ-10), with less differential item functioning and a robust unidimensional factor structure. The ISAAQ-10 severity score correlated strongly with established measures of internet addiction (Compulsive Internet Use Scale [Person's *r* = 0.86] and the Internet Addiction Test-10 [*r* = 0.75]). Combined with gaming activity score it correlated moderately strongly with the established Internet Gaming Disorder Test (*r* = 0.65). Exploratory cluster analyses in both samples identified two groups, one of “low-PUI” [98.1–98.5%], and one of “high-PUI” [1.5–1.9%]. Multiple facets of internet activity appeared elevated in the high-PUI cluster.

**Discussion:**

The ISAAQ-10 supersedes the earlier longer version of the ISAAQ, and provides a useful, psychometrically robust measure of PUI severity (Part A), and captures the extent of engagement in a wide gamut of online specific internet activities (Part B). ISAAQ-10 constitutes a valuable objective measurement tool for future studies.

## Highlights

• ISAAQ was developed to quantify Problematic Usage of the Internet (PUI).
• We refined it to generate a shorter, psychometrically optimized, version (ISAAQ-10).
• The ISAAQ-10 can quantify not only overall severity of PUI but also the nature of online activities.
• Future work could focus on validating the ISAAQ-10 in other languages.

## 1 Introduction

Problematic usage of the internet (PUI) [1] (also referred to by some as Generalized Internet Addiction [2]) continues to be a global concern, with public health implications and growing societal costs [3]. One of the key priorities in PUI research continues to be achieving a reliable conceptualization of PUI [1,3]. Most facets of PUI, apart from online gaming and gambling, are not included in the latest version of the international classification of disease (ICD-11) [4]. PUI nosology, subcategories and causative mechanisms are still under debate and theoretical models continue to evolve in an attempt to capture the newest available evidence [5]. The way PUI is objectively measured has direct impact on the conceptual understanding and neurobiological and clinical determinants of PUI as a nosological construct. Without being able to reliably measure both the overall severity of PUI and the nature of individuals' problematic online behaviors, it is unlikely that the field will advance significantly.

PUI describes the presence of marked functional impairment and/or distress, driven by excessive online activities. Those online activities may be characterized by addictive, impulsive and/or compulsive features, which fuel the persistence of those problematic behaviors, or may represent key vulnerability markers [[6], [7], [8], [9], [10]]. During the COVID-19 pandemic, there was increasing concern over the rising identification of PUI [11]; while the long-term effects of the COVID-19 pandemic on PUI are still unclear, a number of studies indicated a rising prevalence of behavioral addictions during this time [12].

PUI activities can be pleasurable/rewarding, and theoretically mimic (or share similarities with) the effects that addictive drugs can have on the brain's reward circuitry [10]. There have been a number of putative online activities that, if they become addictive (e.g. specific internet addictions [12]), may fall under the umbrella of PUI [1,13,14], such as general *surfing* (unstructured online browsing), gaming, gambling, cybershopping, pornography/cybersex, use of social networking sites (SNS), cyberchondria (over-consumption of health resources), cyberbullying perpetration, streaming media, among others [1,[15], [16], [17]]. These multiple internet-based activities often co-exist and may independently predict the presence of PUI [15]. Other research has highlighted the increasingly overlapping nature of those activities, for example with the ‘gamblification of gaming’ [18], the ‘gamification of cybersex’ [19] or cyberbullying on social media [20]. At the same time, human engagement with the online medium has changed dramatically over the last three decades, and will continue to do so, rendering the characterization of PUI and its determinants an extremely difficult task [21].

Given that PUI is such a wide and complex issue, it is not surprizing that instruments often fail to capture the construct holistically, or strategically choose a narrow focus (e.g. to measure one specific online behavior in isolation, like gaming [22]). Instruments have thus far been optimized and validated mainly for gaming disorder (e.g. the Internet Gaming Disorder Test, IGDT [23]). Instruments designed to measure aspects of generalized PUI also exist – the most psychometrically studied to date being scales such as the Compulsive Internet Use Scale (CIUS) [24], the Problematic Internet Use Questionnaire (PIUQ-9) [25], or the shortened version of Internet Addiction Test (IAT-10) [26,27]. Advancing on these scales, the Assessment of Criteria for Specific Internet-use Disorders (ACSID-11) provides comprehensive coverage of different internet activities (comprising up to 115 questions if all behaviors are endorsed) and has a four factor solution [28].

Recently, we developed a new scale, the Internet Severity and Activities Questionnaire (ISAAQ) [29], which differs from extant scales in several ways. Firstly, ISAAQ was designed not only to capture the overall severity of PUI conceptualized as a unidimensional quasi-trait (Part A of the instrument) [30] but also the extent of engagement in various specific online activities (Part B of the instrument). Secondly, ISAAQ includes questions based on a framework that includes core features of addiction [5] but also extends into other relevant concepts of impulsivity and compulsivity [1,31], in keeping with more recent comprehensive conceptualizations of PUI as well as comorbidity data [1,3,5]; the latter providing insights on the neurobiological commonalities between PUI and disorders of the impulsive-compulsive spectrum.

### 1.1 Aims and objectives

In this study, our primary objective was to psychometrically refine and validate Part A (severity items) of the ISAAQ, towards identifying severity of PUI, using cross-sectional datasets from two different cultural and geographical settings. Our hypothesis was that Part A of the scale could be shortened and its psychometric properties improved using Item Response Theory (IRT). IRT allows for empirically modeling item level data with respect to how they measure an underlying trait, making IRT a useful family of methods for refining existing psychopathological scales [32,33]. We also predicted its properties would be reproducible across the two independent datasets. Our secondary objective was to explore the activities component of ISAAQ (Part B) to gain insights on how various online activities cluster in groups and overlap between each other. We hypothesized that online activities would form data-driven clusters in multidimensional space, indicating affinity between specific activities for users that are allocated within the cluster. Again, we predicted findings would be reproduced across the two independent datasets.

## 2 Material and methods

### 2.1 Study criteria and recruitment

The study used two distinct samples: one in South Africa (SA sample, final sample size with complete scores and demographics *N* = 3275), recruited using convenience and snowballing sampling (more details about recruitment of the SA sample is presented in previous work [34]); and a second sample from USA and the UK (USA-UK, complete sample, *N* = 943) recruited using the Prolific (www.prolific.co) online recruitment platform. Surveys were implemented using Qualtrics. Criteria for inclusion in the study were 1) the ability to undertake the study procedures 2) access to the internet and 3) the ability to provide informed consent. The SA sample included adults aged 18–65 years, whereas the age range of participants in USA-UK sample ranged from 18 to 30 years. To boost recruitment, SA participants had the option to be entered into a prize draw (worth 1000 ZAR, [equivalent to ∼£50]). The USA-UK participants were each compensated with a £10 equivalent. The SA data collection took place from March 26th through to October 2020, and the USA-UK sample from May 12th through December 1st 2021.

### 2.2 Ethical considerations

This study was approved by the Health Research Ethics Committee at Stellenbosch University prior to commencement (SU IRB reference number N19/07/079) for the SA recruitment, and by the Cambridge Psychology Research Ethics Committee (IRB reference number: PRE.2020.141) for USA-UK recruitment. All data collected were kept in secure servers to maintain confidentiality, curated to remove personal identifiable data and no individual responses were accessible beyond the research team. The authors assert that all procedures were conducted according to the guidelines of the Declaration of Helsinki. Participants confirmed consent to partake in the online survey after reading the information about the study.

### 2.3 Demographics assessment

Participants completed demographic details including age, ethnicity and biological sex and gender.

### 2.4 Behavioral assessments

#### 2.4.1 Internet severity and activities addiction questionnaire (ISAAQ)

The Internet Severity and Activities Addiction Questionnaire (ISAAQ) is a two-part questionnaire designed to measure severity of internet addiction (15-item ISAAQ Part A or severity component) and a compendium of putatively problematic internet activities (ISAAQ Part B or activities component) to measure the extent of engagement in online activities respectively, using a 6-point Likert scale (0 = “*Not at all*” to 5 = “*All the time*”). Full questionnaire items can be found in the supplementary file in Table S1 and Table S2.

#### 2.4.2 Established measures of internet use and internet gaming disorder

The survey at both sites included the latest refinement of the Internet Addiction Test (IAT-10) [26], a shortened ten-item version of Young's Internet Addiction test [35] with improved psychometric properties through IRT. The Internet Gaming Disorder Test (IGDT) [23], an established 10-item instrument measuring online gaming that operationalizes the nine DSM-5 criteria for Internet gaming disorder [36], was also included in the survey. The South Africa survey additionally included the short (5-item) Compulsive Internet Use scale (CIUS) [37], an established screening instrument of PUI with very similar sensitivity and specificity to the original full CIUS scale [24].

#### 2.4.3 Behavioral traits with theoretical links to PUI

Impulsivity and compulsivity are important theoretical determinants of PUI [1,3,5,38]. Both sites used the 20-item short Impulsive Behavior Scale Urgency, Premeditation (lack of), Perseverance (lack of), Sensation Seeking, Positive Urgency, Impulsive Behavior Scale (S-UPPS-P), to capture impulsiveness traits [39]. The Chicago-Cambridge Trait Compulsivity Test (CHI-T) [40] was used to capture compulsivity traits in the study population. This is a 15-item instrument comprising two factors, “perfectionism” and “reward drive”, with these factors being previously validated using exploratory structural equation modeling at extremely large population scale [41].

### 2.5 Quality of life assessment

We used the Brunnsviken Brief Quality of Life Scale (BBQLS) [42] to measure self-reported quality of life in both surveys.

### 2.6 Statistical analysis

Statistical analysis was conducted in R statistical software (R version 4.2.1) using R packages “tidyverse” [43], the “mirt” package [44] for IRT and the “lordif” package [45] for Logistic Ordinal Regression Differential Item Functioning (DIF). We used the “cluster” package [46] for all types of exploratory cluster analysis, and the “lavaan” package [47] for confirmatory factor analysis (CFA). Other libraries used included “purrr”, “ggplot2”, “fmsb”, “Bayesrel”, and “gridExtra”. The R code can be provided upon reasonable request to the first author.

#### 2.6.1 Item response theory (IRT)

Polytomous item data were fitted using the graded response [GR] model [48]. For each item, the GR model estimates: 1) a slope parameter (α); and five threshold parameters (β), (one less than the responses on the ISAAQ Likert scale). Threshold parameters reflect the location on the distribution of the underlying trait where the response is most likely to be endorsed and the item is most precise [33]. Slope parameters indicate capacity to discriminate between different levels of the latent trait. Unidimensional IRT models were fitted to explore the scale and item level characteristics of the ISAAQ severity component (Part A: 15-items). Item level fit was assessed with the S-X2 as primary fit index [49], with significant probability values *p* < 0.01 indicating that the observed response patterns do not conform to those predicted by the model. We used the index–S-X2 as primary item fit index [49], which in the “mirt” package calculates a root mean square error of approximation (RMSEA) value and is specifically designed to assess item fit for response models for polytomous ordinal data [50]. We then assessed overall IRT model fit using the M2 index, as well as the Standardized Root Mean Squared Error (SRMR), Tucker Lewis Index (TLI) and comparative fit index (CFI). Items were examined in terms of the Item characteristic curves (ICC), their Item Information Curves (IIC) and their DIF. Slope and threshold parameters were used to generate item characteristic curves (ICCs, also known as Item Response Functions) for the polytomous data, which are a graphic representation of the probabilities of endorsing each item response category across the underlying latent trait continuum theta (*θ,* standardized) [51]. Generally, items with steep and non-overlapping ICCs provide more discrimination across the latent trait. Item parameters can also be used to form item information curves (IICs or item information functions [IIFs]), which indicate the degree of information each item additively contributes at various levels of θ [51]. Item information added together creates the total information function (TIF), which represents the combined measurement precision of items included in the model across the latent trait continuum [33,51]. Evaluating the slope and threshold parameters, as well as inspecting the ICCs, IICs, and TIFs can be helpful to determine the relative impact of removing items from a scale on the overall precision across the latent trait continuum [52].

An iterative approach based on the South Africa dataset, (*n* = 3275) was implemented by examining threshold and slope parameters for each item, as well as the ICCs, IIC and DIF results in which a step-wise elimination of poorly performing items. This led to progressively shorter versions of ISAAQ with less items and new scalar characteristics (from 15-items to 8-items versions). IRT scalar characteristics were examined, including scale information and conditional standard errors, conditional reliability (CR), single IRT reliability estimate (*r*_*xx*_) as well as the scales' characteristic curve (SCC).

DIF analysis is a form of testing of measurement invariance (as in Confirmatory factor analyses, CFA) for IRT [53]. DIF involves the evaluation of conditional relationships between item response slope and threshold parameters and group membership. Our DIF was tested against demographic parameters: age (as numeric, above or equal, and below 25 yrs), gender (Female or Male) and ethnicity (Caucasian or non-Caucasian). Our DIF analysis used logistic ordinal regression with Monte Carlo simulations over 100 replications to flag items for uniform (same across *θ*) and non-uniform (not same across *θ*) DIF. We used alpha <0.001 threshold to reduce false positive discovery due to multiple testing and a relatively large sample [54]. DIF was examined on the level of individual level functioning as well as on a scale level (i.e. Differential Test Functioning or DTF) [52]. The process was repeated on the models with decreasing number of items using an iterative approach. Our analyses examined the SA and the USA-UK samples separately. The larger SA sample was used as reference sample in the iterative approach. The USA-UK sample was tested second as focal sample to ascertain the replicability of the results.

#### 2.6.2 Confirmatory factor analysis for the IRT recommended model

The IRT-based iterative approach that we followed indicated that a shorter version of ISAAQ may have improved IRT properties. We performed EFA to establish essential unidimensionality and followed this up with a unidimensional CFA to establish the shorter scale's psychometric properties. Our CFA used a Diagonally Weighted Least Squares (DWLS) estimation with Nonlinear Minimization subject to Box Constraints optimization method [55]. DWLS is specifically designed for ordinal data and makes no distributional assumptions about the observed variables; however, it assumes a normal latent distribution underlying each observed variable. We examined the residuals covariance matrix and modelled residual correlations above ±0.1, as likely representing common wording or item context effects. We calculated fitness metrics for the final CFA model, including, Comparative fitness index (CFI), Tucker-Lewis Index (TLI) and Standardized Root Mean Square Residual (SRMR), Root Mean Squared Error Approximation (RMSEA).

#### 2.6.3 Validity metrics for the IRT recommended model

We calculated the scale's internal consistency by calculating Cronbach's alpha and Guttman's lambda, with coefficients above 0.8 indicating good internal consistency. Also, convergent validity by calculating its Pearson's *r* correlations with established measures of internet use (IAT10, CIUS) and gaming disorder (IGDT) as well as further construct validity by calculating correlations with other known determinants of PUI (e.g., impulsivity, using S-UPPS-P 5-factors, and compulsivity using CHI-T 2-factors), as well as quality of life measure (using BBQLS total score). A value of |r| = 0.10, 0.30, 0.50 indicating a small, medium, large effect, respectively.

#### 2.6.4 Exploratory cluster analyses

We used cluster analyses to examine the activities component of ISAAQ (Part B). Our analyses examined the SA and the USA-UK samples separately to ascertain replicability of clusters across samples/cultures. We explored the appropriate number of clusters by consulting the standard methods of an elbow-plot [56] and silhouette plot [57] (elbow and silhouette plots are presented in the supplemental Figs. S3 and S4). The elbow plot examines total distance across all levels of k-means clustering and silhouette plot examines silhouette scores (high levels of silhouette indicating preferred number of clusters). Given the high number of existing methods to determine the number of clusters, additionally to the elbow and silhouette plots, we used the “Nbclust” set of methods to test 26 methods of determining the number of complete linkage hierarchical clusters and used majority vote. We then used hierarchical clustering with complete linkage on the chosen number of clusters to create the clustering assignments, which although is more computationally demanding to k-means, can provide replicability in the clustering assignments for each group.

## 3 Results

### 3.1 Descriptive statistics

Descriptive statistics of the two samples are presented in detail in the online supplement (see supplemental Table S3a and S3b). The mean ages for the SA and USA-UK samples were 24.5 and 24.4 years, respectively. The SA sample had a higher percentage of females and non-Caucasian subjects compared to the USA-UK sample (65% vs. 58% (χ^2^, *p* < 0.001) and 57% vs. 29% (χ^2^, p < 0.001) respectively).

### 3.2 Original ISAAQ and ISAAQ-10

IRT parameters were calculated for ISAAQ original (15-items) as well as shortened versions using an iterative approach (described in §2.6.1). The 10-item version (henceforth ISAAQ-10) appeared to be a suitable candidate for further examination, as removing further items (shorter than 10-item versions of ISAAQ) did not further improve IRT DIF metrics and had an undesirable impact on content validity and scale reliability. We henceforth present the original ISAAQ and ISAAQ-10 for reasons of simplicity.

### 3.3 Item characteristic curves

**Fig. 1:**
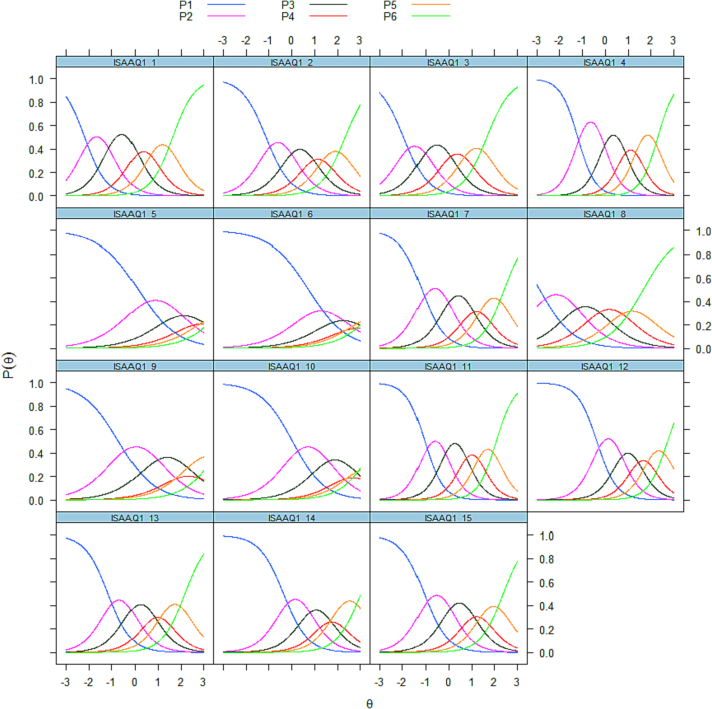
Item characteristic curves. Legend: *Item Characteristic Curves (ICCs) for 15-items on the ISAAQ Part A severity component (SA sample n* *=* *3275). Theta (θ) indicating the ability scores measuring PUI on a unidimensional latent trait. Most items demonstrate good capacity to discriminate levels of θ across the Likert graded response, however, items 5, 6, 9, and 10 appear less so, particularly in the higher levels of θ (+2-3sd). They also had the smaller (less steep) slopes.*

Item Characteristic Curves (ICCs) for 15-items on the original ISAAQ part A severity component (SA sample *n* = 3275) are presented in Fig. 1. Theta (*θ*) indicates PUI represented as a unidimensional latent trait in standardized metric (*M* = 0, *SD* = 1). Most items demonstrated good capacity to discriminate levels of θ across the Likert graded responses, however, items 5, 6, 9, and 10 appeared less able to do so, particularly in the higher levels of *θ* (+1-3sd). We examined slope (alpha parameters) for each item and those items ranked last (by order 5, 6, 9, 8, and 10); slope indicates the ability of the item to differentiate at different levels of the latent trait. Full IRT parameter scores, including slope and threshold are presented in supplemental Table S4.

### 3.4 Item information curves

**Fig. 2:**
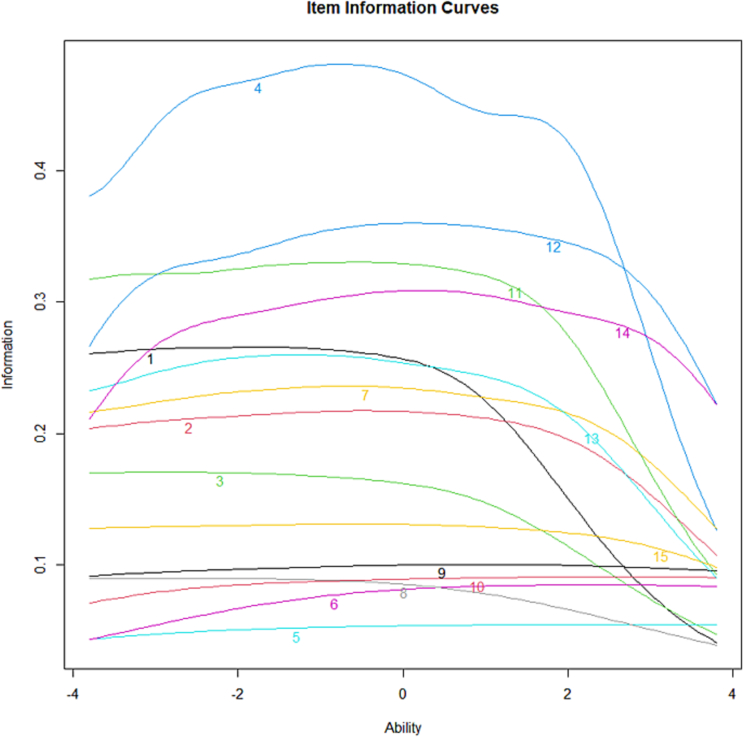
Item information Curves. Legend: *The Item information Curves (IICs or Item Information Functions, IIFs) from the SA sample (n* *=* *3275), 15-items of the original ISAAQ Part A (severity component).*

Item information curves are presented in Fig. 2. Items 5, 6, 8, 9, and 10 appeared to have the lowest item information area across most levels of θ.

### 3.5 Test information function

**Fig. 3:**
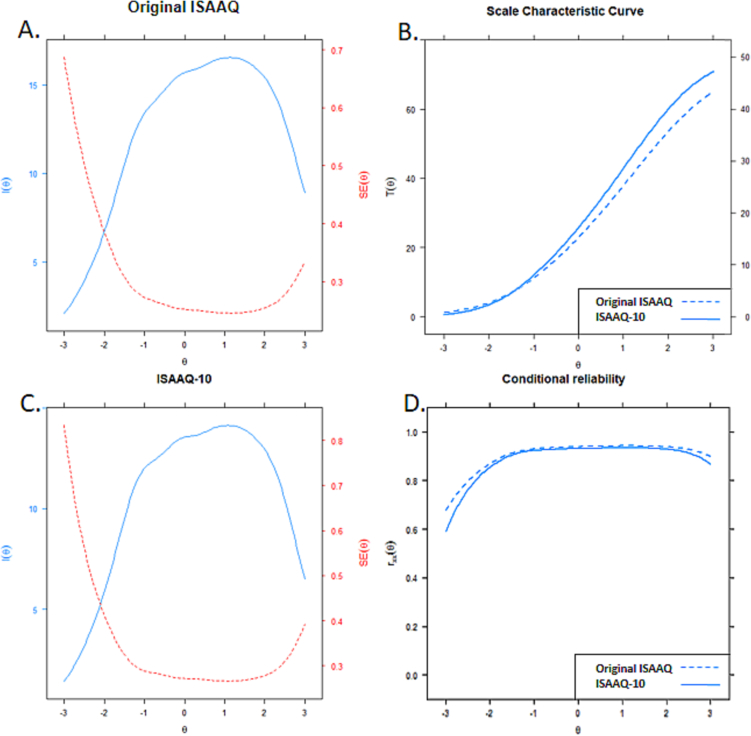
Test information functions with standard errors (A, C, left), scale characteristic curves (B, top right) and conditional reliability curves (D, bottom right) of original ISAAQ and ISAAQ-10. Legend: *Test information function for both the* original *ISAAQ (A, top left) and ISAAQ-10 (C, bottom left). The scale of test information is shown on the on the left side of the y-axes and plotted in blue. The scale of the standard error is on the right side of the y-axes, plotted in red. The standard error is expected to be lower where information is higher. It is typical that the standard error would increase at the extreme ranges of theta (θ) where there is less information. The scale characteristic curves for the original ISAAQ and the ISAAQ-10 are plotted together in top right (B). The conditional reliability of the original ISAAQ and the ISAAQ-10 are plotted together in the bottom right (D).* (For interpretation of the references to colour in this figure legend, the reader is referred to the web version of this article.)

The IIFs can be used to create the Test Information Function (TIF). TIF can be used to judge the test as a whole, but most importantly to identify which parts of the trait range are measured with the greatest precision and therefore it is an essential part of test development. Both the original ISAAQ and ISAAQ-10 had very similar TIFs, with the original ISAAQ having greater information across the latent trait continuum due to having more items. However, both the original ISAAQ and the ISAAQ-10 performed similarly across all levels of the latent trait continuum (See Fig. 3, top left, bottom left). The scale characteristic curves of original ISAAQ and ISAAQ-10 are presented in Fig. 3 (top right). The scales' conditional reliability plots are presented in Fig. 3 (bottom right).

Particularly strong areas for both versions of the scale were those between −1.5 < θ < +2.5, which are essential areas of θ for a severity instrument. Conditional reliability curves were similar between the two versions of the scale.

### 3.6 Differential item functioning

Results from examining differential item functioning (DIF) across three main demographic characteristics (age, sex and ethnicity) are presented in Table 1. The results include an iterative approach by which an item of the severity component of ISAAQ (15-item) is eliminated at each step. For all IRT models in both samples, acceptable IRT model fit metrics were demonstrated, including SRMSR <0.05, TLI >0.95, CFI > 0.95 and RMSEA.S_X2 (item fit) <0.05 in all items. Full IRT metrics for both samples are presented in the supplement (see supplemental TABLE S5 and S6). Item level and scale level DIF exploratory plots are presented in the supplement (supplemental Figs. S1a, S1b, S1c for age, gender and ethnicity, respectively).

**Table 1:** IRT metrics including differential item functioning.

SA sample, *n* = 3275						
	Item drop	Proportion variance	IRT reliability *r*_*xx*_ estimate	DIF flagged items (age, as binary above or below 25 yrs)	DIF flagged items (gender, as binary Female or Male)	DIF flagged items (ethnicity, as binary Caucasian or non-Caucasian)
Original ISAAQ (15-items)	Null	0.52	0.93	1, 4, **5**, **6**, 7, **9**, 13, 15	2, **5**, **6**, 7, **8, 10**, 11, 12	1, 2, **6, 8, 9**, 12
ISAAQ-10	5, 6, 8, 9, 10	0.61	0.92	1, 3, 7, 13	2, 7, 11, 12, 13	1, 2, 12
USA-UK sample, *n* = 943						
Original ISAAQ (15-items)	Null	0.46	0.91	1, 2, **5**, 13	1, 2, **6, 8, 10**, 12, 15	**8, 10**
ISAAQ-10	5, 6, 8, 9, 10	0.51	0.90	1, 13	2, 12, 15	null

Legend: *Item Response Theory (IRT) metrics, including differential item functioning (DIF). Item drop = ISAAQ item removed at each iteration; Proportion variance = proportion of item level variance explained by the latent trait; r*_*xx*_ *= IRT scale reliability estimate; DIF = Differential Item Functioning.*

Items with DIF were prioritized to be removed at each iteration. The ISAAQ-10 had significantly less DIF flagged by the Monte Carlo thresholds compared to the original ISAAQ. In this analysis <50% of items were flagged in all tests of ISAAQ-10. Upon examining each item individually, no item-level DIF had any impact on the DTF, suggesting that the ISAAQ-10 on a scale level performs similarly across the tested demographic groups (age, gender and ethnicity) (See DIF figures in supplement Fig. S1a, S1b, S1c).

### 3.7 Confirmatory factor analyses and validity metrics

Internal consistency was tested with Cronbach's alpha (ISAAQ-10, α = 0.92 (0.916–0.924)) and Guttman's lambda-2 (ISAAQ-10, λ_2_ = 0.92 (0.916–0.924)). EFA showed essential unidimensionality for both original ISAAQ and ISAAQ-10 (see supplemental figs. S2). We performed two CFAs, one in the original 15-item severity ISAAQ and one to examine the properties of the 10-item ISAAQ severity component. Standardized item factor loadings in the two versions of the scale are presented in Table 2 below. The five items which were identified by the iterative IRT approach above were also the lowest loading items among all original ISAAQ items. This finding aligns with the decision to remove those items from the scale, to improve the scale's psychometric properties when aiming to measure a unidimensional construct. They also support the unidimensional IRT model assumptions.

**Table 2:** Standardized item Factor loadings in the two versions of the scale.

	15-item ISAAQ	10-item ISAAQ
Item 1	0.73	0.79
Item 2	0.71	0.69
Item 3	0.70	0.69
Item 4	0.80	0.80
Item 5	0.49*	–
Item 6	0.48*	–
Item 7	0.72	0.70
Item 8	0.59*	–
Item 9	0.57*	–
Item 10	0.56*	–
Item 11	0.79	0.76
Item 12	0.74	0.73
Item 13	0.73	0.74
Item 14	0.68	0.69
Item 15	0.70	0.67

Legend: Standardized item factor loadings in the ISAAQ severity component, 15-items and 10-items. *items that were dropped in the 10-item version. They are also the lowest loading items in the original scale.

We also report standardized fitness metrics for CFA in the two scale versions. Details are presented Table 3. Both versions of ISAAQ had good fitness metrics.

**Table 3:** CFA fitness metrics.

	*df*	χ^2^	*p*-value	RMSEA	CFI	TLI	SRMR
original ISAAQ	83	285.271	<0.001	0.028–0.029	0.99	0.99	0.033
ISAAQ-10	33	126.422	<0.001	0.030–0.030	0.99	0.99	0.018

Legend: *Data in this table are from the SA sample. ISAAQ = Internet Severity and Activities Addiction Questionnaire; df = degrees of freedom; **χ***^***2***^ *= chi square statistic; RMSEA = Root Mean Squared Error Approximation, (Bootstrap 90% confidence interval from 1000 iterations); CFI = Comparative Fit Index; TLI = Tucker-Lewis Index; SRMR = Standardized Root Mean Squared Error.*

### 3.8 Construct validity

We also calculated Pearson's correlation coefficients between ISAAQ, IAT10, CIUS and IGDT as convergent validity metrics (see Table 4). Internal consistency metrics were calculated for all instruments (CIUS, Cronbach's α = 0.80, McDonald's ω = 0.80; IAT10, α = 0.82, ω = 0.83; IGDT, α = 0.89, ω = 0.90; S-UPPS-P, α = 0.80, ω = 0.80; CHI-T, α = 0.78, ω = 0.78; BBQLS, α = 0.80, ω = 0.80). Both versions of ISAAQ correlated moderately strongly with established measures of PUI [for original ISAAQ: ∼IAT10, *r* = 0.75 (0.73–0.76, *p* < 0.001); ∼CIUS, *r* = 0.86 (95%CI: 0.85–0.87, p < 0.001); for ISAAQ-10 ∼ IAT10, *r* = 0.71 (0.69–0.73, p < 0.001); ∼CIUS, r = 0.86 (95%CI: 0.85–0.87, p < 0.001)] indicating convergent validity. In examining convergent validity with internet gaming disorder, we used the simple ISAAQ severity score, which correlated moderately with IGDT, however the latter measures internet gaming disorder, not PUI. Thus, we used a composite score of [ISAAQ (severity score) × ISAAQ (Gaming, Activity score)] and that correlated moderately strongly with the total IGDT score. For further construct validity, we calculated correlations between ISAAQ and behavioral traits of impulsivity (S-UPPS-P) and compulsivity (CHI-T). The results suggested that the ISAAQ correlates well with theoretical determinants of PUI [5,31] (see Table 4). Finally, we examined correlations between the ISAAQ and the BBQLS. The inverse correlation (*r* = −0.22; 95%CI (−0.18 to −0.26), *p* < 0.001) suggested that participants with higher levels of PUI have been experiencing lower levels of quality of life, adding to the construct validity of ISAAQ-10. Differences in construct validity metrics between the original ISAAQ and ISAAQ10 were negligible. Full construct validity results are presented in Table 4 below.

**Table 4:** - Construct validity metrics for original ISAAQ vs ISAAQ10 in the South Africa sample.

	Internal consistency metrics (Estimates of Single-Test Reliability Measures)			Construct validity metrics / Persons correlations					
	Cronbach's α	Guttman's λ	Omega ω	IAT10*	CIUS	IGDT**	S-UPPS-P	CHI-T	BBQLS
**original ISAAQ**	0.92–0.93	0.92–0.93	0.92–0.93	0.75	0.86	0.41 / (0.65)	NU = 0.43 PU = 0.36 SS = 0.06 LPM† = −0.23 LPS† = −0.17	PER = 0.16 RD = 0.51	−0.22
**ISAAQ-10**	0.915–0.924	0.916–0.924	0.916–0.924	0.71	0.86	0.39 / (0.65)	NU = 0.43 PU = 0.34 SS = 0.06 LPM† = −0.22 LPS† = −0.19	PER = 0.16 RD = 0.51	−0.22

Legend: Data in this table are from the SA sample. ISAAQ = Internet Severity and Activities Addiction Questionnaire; IAT10 = Internet Addiction Test, 10-items; CIUS = Compulsive Internet Use Scale; IGDT = Internet Gaming Disorder Test; S-UPPS = The Short Urgency, Premeditation (lack of), Perseverance (lack of), Sensation Seeking, Positive Urgency, Impulsive Behavior Scale; NU = Negative urgency; PU = Positive urgency; SS = Sensation seeking; LPM = lack of premeditation; LPS = lack of perseverance; CHI-T = Chicago-Cambridge Compulsivity Trait Scale; PER = Perfectionism; RD = Reward Drive; BBQLS = Brunnsviken Brief Quality of Life Scale;*Correlations with IAT-10 used mean imputation on missing data (n = 388), correlations without imputation were higher to those reported in the table i.e. IAT-10 ∼ orignial IAT r = 0.81, ISAAQ-10 ∼ IAT-10 r = 0.76; **the number in brackets indicate the Pearson's correlation between IGDT score and the ISAAQ gaming score = the severity component total multiplied by the Gaming activity score (ISAAQ Part B), due to missing data in IGDT (n = 178) mean imputation was used. Correlations without data imputation had negligible differences to those presented in the table; † those items are inversely coded.

### 3.9 Exploratory cluster analysis of the ISAAQ activities component

**Fig. 4:**
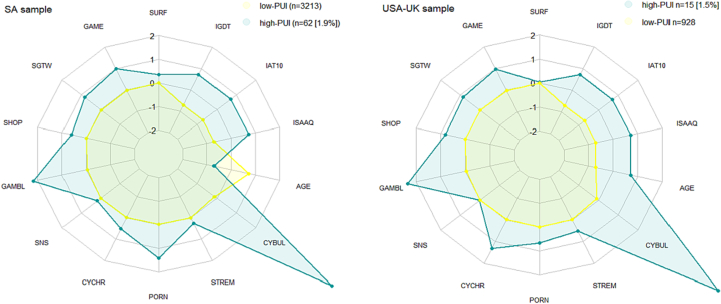
Radar plots. Legend: *Radar (spider) plot with colour indicators from the clustering groupings. Yellow colour = non-problematic internet users; Petrol colour = Problematic internet users; Scores indicate internet activities from ISAAQ activities component (Part B), from the SA sample (left, n* *=* *2910) and the USA-UK sample (right, n* *=* *943). Plot uses standardized measures for all activities and scales. Left plot = South Africa sample, Right plot = USA-UK sample. SURF = General surfing; GAME = Online gaming; SGTW = Skill games and time wasters online; SHOP = Online shopping; GAMBL = Online gambling; SNS = Online social media use; CYCHR = Cyberchondria; PORN = Online pornography use; STREM = Online Streaming; CYBUL = Cyberbullying (perpetration); AGE = participant age; ISAAQ = ISAAQ-15 scores; IAT10 = Internet Addiction Test 10-item score; IGDT = Internet Gaming Disorder Test score. Spider homocentric polygons demonstrate standardized lines scores (from -2sd to +* *2sd).* (For interpretation of the references to colour in this figure legend, the reader is referred to the web version of this article.)

Exploratory clustering diagnostics (see elbow and silhouette plot in supplemental Figs. S3 and S4) to guide number of clusters using the ISAAQ activities component (Part B) supported a two cluster solution. Further diagnostics across 26 “NbClust” methods to determine number of clusters, the best number of clusters was two in both the SA and USA-UK samples. Full details of “NbClust” diagnostics are presented in supplemental paragraph §S1. Due to not-at-random data missingness, mean value imputation was used (for IAT10 *n* = 338; IGDT, *n* = 179; GAMBL, n = 1; CYBUL *n* = 2; STREM, n = 1; PORN, n = 1). Contrary to our hypothesis, hierarchical cluster analyses showed that specific online activities did not cluster together, but rather indicated that all activities tended to co-occur at different levels of severity. Those clusters were also characterized by distinct levels of generalized internet usage (indicated by elevated measures of ISAAQ severity score, IAT10 and IGDT scores), despite the fact that no overarching PUI metric was used in the clustering process. The clustering was almost exactly replicated in the USA-UK sample, with the clustering process identifying two clusters of very similar characteristics in terms of size and level of activities (see Fig. 4). Based on inspection of the characteristics of the cluster samples, they were henceforth labelled as ‘high-PUI’ and ‘low-PUI’. The high-PUI cluster (1.5–1.9% of the total sample) comprised individuals with high levels of PUI and gaming addiction, as well as high engagement in multiple facets of online usage (i.e. gaming, gambling, shopping, cyberbullying, pornography etc.). Results from the standardized scores across each online activity are presented in graphic form in two separate radar plots (see Fig. 4). Exact scores for each online activity and cluster groups are presented in the online supplement (see supplemental Tables S7 and S8).

The similarities between the two samples in this clustering process are supportive of similar responses across culturally and geographically distinct sites.

## 4 Discussion

### 4.1 The optimization and validity of the ISAAQ severity component

In this work we psychometrically refined the original ISAAQ (15-item scale) [58] Part A severity component by examining the IRT properties of the individual items and removing low performing items using an iterative approach. The shorter, validated ISAAQ-10 severity component demonstrated similar psychometric properties (e.g., similar IRT reliability, test information function) to the full-length instrument, but with fewer items demonstrating undesirable differential item functioning. The ISAAQ-10 also demonstrated strong psychometric properties, including unidimensionality, excellent internal consistency reliability, and convergent and criterion validity. Furthermore, another positive feature was that there was no evidence of differential test functioning of the ISAAQ-10 across age, sex and ethnicity groupings, supporting its use across diverse samples.

The ISAAQ-10 mapped well, in terms of correlations, onto two widely used PUI instruments – the CIUS and the IAT-10. However, in contrast to these instruments it offers potential advantages: it maps not only severity (Part A; with a robust unifactorial solution) but also measures the range of individual online activities (Part B). Furthermore, the activities compendium can be used in conjunction with the severity component to provide a valid measure in a particular area of PUI (e.g., in this study ISAAQ-10 severity × Gaming Activity severity correlated excellently with IGDT (*r* = 0.74), the latter being regarded by many as the current ‘gold standard’ for assessing gaming disorder). The ISAAQ Part B activities list can be adjusted easily, i.e., adding/removing specific activities which may be a focus area in a specific line of research. For example, a previous study used an alternative 12-item version of the ISAAQ activities component (Part B) to capture consumption of sports-related content and digital-hoarding [59]. The same principle can apply to other areas of research, where a specific focus on another online activity is needed (e.g., dating Apps, calorie-tracking Apps, or cyberbullying victimization) [60]. This can provide a reasonable solution for research studies which aim to capture a wide range of online activities at the same time, in an efficient manner, without jeopardising the objective quantification of overall PUI severity, which is captured separately in Part A of the tool (Part A should not be modified). Future research can build on this study to provide validation of the ISAAQ severity × activities scores, by examining how those correlate with other more extensive questionnaires that focus on a specific area of online use, such as the Bergen Social Media Addiction scale [61] or the different subcomponents of the ACSID-11 [28].

### 4.2 Exploratory cluster analysis

The exploratory cluster analysis of the ISAAQ activities component (part B) supports the notion that PUI activities overlap and co-exist within the higher levels of the PUI latent trait, adding to the importance of considering them together when measuring PUI across multiple activities. Previous work from our group showed that different online activities, when considered together, can independently statistically predict PUI (by virtue of out-of-sample cross-validated LASSO regression), supporting the notion of PUI as a multifaceted concept [15]. We also previously showed that PUI clusters across activities (i.e., there are no subtypes based on online activity) [30]. The new advances in understanding PUI continue to stress the importance of understanding PUI across a wide range of activities [1,3].

Building on these findings, another interesting result from the clustering analysis was that the presence of cyberbullying (perpetration) behaviors almost exclusively existed within the “high-PUI” cluster, in both SA and USA-UK samples independently. This aligns with the notion that cyberbullying behaviors co-exist and overlap with other online activities happening to a problematic degree and it might be helpful for them to be understood in that context. Other approaches exclude cyberbullying perpetration from the PUI umbrella (e.g. see [17]), given that there might be different psychological mechanisms underpinning those behaviors (e.g. conduct problems or anti-social personality). However, in our view, capturing the overlap of those behaviors with other PUI activities is important (e.g., arguably much of cyberbullying happens on social media [20] or during multiplayer gaming or cyber-harassment linked to pornographic online content [62]).

### 4.3 Limitations

There are a number of limitations to be considered. First, the datasets in this study were collected online. Online surveys were the norm for research that happened during the COVID-19 pandemic. Online survey methods offer clear advantages in terms of scalability coupled with low risk of transmission of infection during pandemics; however, they have diminished accuracy for measuring psychopathology constructs as compared to face-to-face clinical assessments. At the same time, the large scale nature of the datasets renders this less problematic, and indeed they are a convenient and desirable prelude to conducting face-to-face clinical assessments in future work. Another limitation comes from the snowball and convenience recruitment used in the SA sample, which may limit the generalizability of results. The USA-UK samples used Prolific, which offers a more standardized/stratified approach to recruitment and another source for testing the replicability of results. A final limitation comes from the fact that data was collected during the COVID-19 pandemic during which there was potentially higher frequency of usage of online technology. While this may theoretically inflate the measured point prevalence of problematic online behaviors the levels of PUI severity measured in this study were comparable to the reported pre-pandemic levels locally and globally [2,30].

### 4.4 Future research directions

Now that the ISAAQ has been psychometrically refined and validated, a next step could be to identify and validate useful ‘caseness thresholds’ using the scale measured against rigorous in-person clinical interviews using structured instruments and incorporating measures of functional impairment. Such work would require testing the instruments classification metrics (e.g., sensitivity, specificity etc.). For some activities, such as online gaming, this could be done against current ICD definitions. For other activities, many of them do not have set diagnostic criteria yet and remain under consideration for inclusion in the diagnostic classification manuals or continue to be explored in terms of their theoretical basis and nosology [3,4,13,17,63].

We found that the ISAAQ-10 performed best, in terms of TIF, between −1.5 < θ < +2.5 (PUI as unidimensional latent trait), which are essential areas of θ for a severity instrument in the general population. It is unclear whether the lower level of performance at the extreme upper end, which would be valuable to identify the extreme upper end of severity, is due to the scale not being able to identify that, or due to the sample specifically, which may have contained only a small number of severe cases. Future work can address this by examining a higher percentage of participants on the extreme end of PUI. The performance of the scale in the lower end of θ (e.g., less than −1.5) is less critical for the value of the scale, due to the fact that this range is of less critical importance for severity or clinical screening and PUI has been theorized as a unidimensional quasi-trait, with the vast majority of meaningful variance on the upper level of θ [30].

Crucial steps for future research could also include a focus on identifying vulnerability and chronicity predictors for PUI, by conducting large-scale longitudinal research. Such research would ideally need to combine such validated measures with scalable online assessment platforms (ideally also measuring cognitive functions implicated in PUI).

## 5 Conclusion

We have provided evidence that the ISAAQ-10 is a shorter, valid, and useful measure of internet use, measuring severity unidimensionally, but also the extent of engagement in various types of online activity. The severity scale (Part A) together with the activities list (Part B) can provide useful insights across a wide range of specific internet activities. Furthermore, it can offer the necessary flexibility that is required to capture the very complex and quickly changing nature of PUI, since activities can be added or removed from Part B without impacting the overall severity measure that is captured by Part A. The preliminary cluster analysis of the activities component supports the notion that PUI activities overlap and co-exist within the higher levels of the PUI latent trait, adding to the importance of considering them together when measuring PUI across multiple activities.

## Author contribution

KI, analyzed the data, coordinated authors contributions and led on drafting the manuscript. SRC, JEG and CL designed the study protocol and were lead investigators. CO, CL and NL conducted the data collection and initial curation. JT provided support with analysis and results interpretation. All authors contributed to the writing of the manuscript and approved the final version for submission.

## Funding

This research was funded in whole, or in part, by the National Research Foundation of South Africa (Ref 118567) to CL and 10.13039/100010269Wellcome Trust [110049/Z/15/Z & 110049/Z/15/A] to SRC. Additional funding came from internal funds from the 10.13039/100007234University of Chicago provided by JEG. SRC's role in this study was funded by a Wellcome Trust Clinical Fellowship (110049/Z/15/Z & 110049/Z/15/A). JT was supported by the Turner Impact Fellowship from the Turner Institute for Brain and Mental Health, Monash University. The sponsors, had no role in the study design, in the collection, analysis and interpretation of data, in the writing of the report, and in the decision to submit the article for publication.

## ISAAQ usage permissions / copyright information

The ISAAQ (all versions) are copyright Konstantinos Ioannidis and Samuel R Chamberlain. The original ISAAQ should no longer be used as it is replaced by the ISAAQ-10. The ISAAQ-10 may be used for non-commercial research purposes without permission, as long as any resulting publications or presentations acknowledge the copyright holders and appropriately cite the validation paper(s). To use ISAAQ-10 for commercial purposes, please contact the copyright holders in advance to request a license.

Item-level content of the ISAAQ (all versions) cannot be reproduced, modified, and/or published without prior written permission of the copyright holders.

## Declaration of Competing Interest

SRC receives honoraria from Elsevier for editorial work. JEG reports grants from Janssen and Biohaven Pharmaceuticals and others from Oxford Press, Norton, McGraw-Hill, and American Psychiatric Publishing outside of the submitted work. KI and SRC are copyright holders for the Internet Severity and Activities Addiction Questionnaire (ISAAQ). The other authors declare that they have no conflict of interest.

## Data Availability

The data that support the findings of this study are available on legitimate scientific non-commercial request from the corresponding author, subject to agreement of the Chief Investigator for the respective data set. The data are not publicly available due to privacy or ethical restrictions.

## Appendix A Supplementary data

Supplementary material Image 1
