# Supplementary material for: Internet severity and activities addiction questionnaire (ISAAQ): Psychometrics of item response theory and clustering of online activities
Source: Compr Psychiatry. 2023 Apr;122:152366. doi: 10.1016/j.comppsych.2023.152366 (PMC9993400; doi:10.1016/j.comppsych.2023.152366)

## Table S1 – The Full Original Internet severity and activities addiction Questionnaire, Severity component (Part A). Note this version is outmoded following the current study, but is included for reader information.

| **#** | **Question** | **Rating Scale** | | | | | |
| --- | --- | --- | --- | --- | --- | --- | --- |
|  |  | Not at all | Rarely | Occasionally | Frequently | Very often | All the time |
| 1 | How often do you find yourself loosing track of time while engaging on an internet related activity? | 0 | 1 | 2 | 3 | 4 | 5 |
| 2 | How often do you use internet related activities to block out disturbing thoughts about your life and to soothe yourself? | 0 | 1 | 2 | 3 | 4 | 5 |
| 3 | How often do you choose to spend time on internet related activities to battle loneliness or boredom? | 0 | 1 | 2 | 3 | 4 | 5 |
| 4 | How often do you neglect your normal day-to-day activities to spend more time on an internet related activity? | 0 | 1 | 2 | 3 | 4 | 5 |
| 5 | How often do you find yourself choosing to spend time in an online activity over intimacy with your partner? (NOT IN ISAAQ-10) | 0 | 1 | 2 | 3 | 4 | 5 |
| 6 | How often do you suffer from negative financial consequences because of an online activity? (NOT IN ISAAQ-10) | 0 | 1 | 2 | 3 | 4 | 5 |
| 7 | How often do your school/study suffer because of the amount of time you spend on internet related activities? | 0 | 1 | 2 | 3 | 4 | 5 |
| 8 | How often do you check your email or social media account or equivalent before something else that you need to do? (NOT IN ISAAQ-10) | 0 | 1 | 2 | 3 | 4 | 5 |
| 9 | How often do others in your life complain to you about the amount of time you spend online on your computer, tablet, mobile or similar device? (NOT IN ISAAQ-10) | 0 | 1 | 2 | 3 | 4 | 5 |
| 10 | How often do you become defensive or secretive about your online activities? (NOT IN ISAAQ-10) | 0 | 1 | 2 | 3 | 4 | 5 |
| 11 | How often do you find yourself trying to stop an excessive or repetitive online activity but feeling an urge to continue? | 0 | 1 | 2 | 3 | 4 | 5 |
| 12 | How often do you feel preoccupied with the internet when off-line, or fantasize or get repetitive urges to get on-line? | 0 | 1 | 2 | 3 | 4 | 5 |
| 13 | How often do you lose sleep due to late-night internet related activities? | 0 | 1 | 2 | 3 | 4 | 5 |
| 14 | How often do you find yourself experiencing physical or psychological problems as a consequence of prolonged Internet related activities? | 0 | 1 | 2 | 3 | 4 | 5 |
| 15 | How often do you try to cut down the amount of time you spend online and fail? | 0 | 1 | 2 | 3 | 4 | 5 |

## ISAAQ-10 (Internet Severity and Activities Addiction Questionnaire, 10-items)

| **#** | **Question** | **Rating Scale** | | | | | |
| --- | --- | --- | --- | --- | --- | --- | --- |
|  |  | Not at all | Rarely | Occasionally | Frequently | Very often | All the time |
| 1 | How often do you find yourself loosing track of time while engaging on an internet related activity? | 0 | 1 | 2 | 3 | 4 | 5 |
| 2 | How often do you use internet related activities to block out disturbing thoughts about your life and to soothe yourself? | 0 | 1 | 2 | 3 | 4 | 5 |
| 3 | How often do you choose to spend time on internet related activities to battle loneliness or boredom? | 0 | 1 | 2 | 3 | 4 | 5 |
| 4 | How often do you neglect your normal day-to-day activities to spend more time on an internet related activity? | 0 | 1 | 2 | 3 | 4 | 5 |
| 5 | How often do your school/study suffer because of the amount of time you spend on internet related activities? | 0 | 1 | 2 | 3 | 4 | 5 |
| 6 | How often do you find yourself trying to stop an excessive or repetitive online activity but feeling an urge to continue? | 0 | 1 | 2 | 3 | 4 | 5 |
| 7 | How often do you feel preoccupied with the internet when off-line, or fantasize or get repetitive urges to get on-line? | 0 | 1 | 2 | 3 | 4 | 5 |
| 8 | How often do you lose sleep due to late-night internet related activities? | 0 | 1 | 2 | 3 | 4 | 5 |
| 9 | How often do you find yourself experiencing physical or psychological problems as a consequence of prolonged Internet related activities? | 0 | 1 | 2 | 3 | 4 | 5 |
| 10 | How often do you try to cut down the amount of time you spend online and fail? | 0 | 1 | 2 | 3 | 4 | 5 |

## Table S2 - The Internet severity and activities addiction Questionnaire, Activities component (Part B)

Over the **last 6 months**, I have spent time on **non-work or study** related online activities as such:

|  | INTERNET ACTIVITIES SCALE | Rating Scale | | | | | |
| --- | --- | --- | --- | --- | --- | --- | --- |
| 1 |  | Not at all | Rarely | Occasionally | Frequently | Very often | All the time |
|  | **General Surfing** (includes any unstructured online activities) | 0 | 1 | 2 | 3 | 4 | 5 |
| 2 | **Internet gaming including Massively-Multiplayer-Online-Role-Playing-Games** (includes online gaming and gaming with multiple other players and role-playing format) | 0 | 1 | 2 | 3 | 4 | 5 |
| 3 | **Skill games & Time wasters** (includes games & applications on computer, tablet, mobile phone or similar for which activity is without specific benefit) | 0 | 1 | 2 | 3 | 4 | 5 |
| 4 | **Online Shopping** (includes activity on online shopping platforms and auction websites) | 0 | 1 | 2 | 3 | 4 | 5 |
| 5 | **Online gambling** (includes any online activity in which there is a chance for monetary gain or other stakes) | 0 | 1 | 2 | 3 | 4 | 5 |
| 6 | **Social networking** (includes browsing social media and messaging/communicating over online social platforms) | 0 | 1 | 2 | 3 | 4 | 5 |
| 7 | **Health & medicine** (includes any online activity relating to reading & researching medical facts, diagnoses, treatments and risks) | 0 | 1 | 2 | 3 | 4 | 5 |
| 8 | **Pornography** (includes cybersex, cyber-texting, viewing pornography and other online sexual activities) | 0 | 1 | 2 | 3 | 4 | 5 |
| 9 | **Streaming media** (include music or video streaming activities on any platform) | 0 | 1 | 2 | 3 | 4 | 5 |
| 10 | **Cyberbullying** (includes exchange of insults, nasty texts/emails, unpleasant media, pranks) | 0 | 1 | 2 | 3 | 4 | 5 |

## Table S3a - Demographics and descriptive statistics

|  | **South Africa** Mean (sd) or [%] | **USA-UK** Mean (sd) or count[%] |
| --- | --- | --- |
| **Age** | 24.5 (27.4) | 24.4 (3.6) |
| **Gender** | Male = 1279  Female = 2351  Non-Binary / Non-specified = 18  After exclusion of non-binary and missing values, used in DIF  F M  2129 [65%] 1146 [35%] | Male = 399  Female = 544  Non-binary/ Non-specified = 17  After exclusion of non-binary and missing values, used in DIF  F M  544 [58%] 399 [42%] |
| **Ethnicity** | Black = 1303  Coloured/Mixed = 509  Indian = 369  White/Caucasian = 1522  Other = 45  After exclusion of missing values, used in DIF  Caucasian non-Caucasian  1410 [43%] 1865 [57%] | Asian = 124  Black, African, or Caribbean = 61  Hispanic or Latino = 51  Middle Eastern = 3  Mixed/Multiple ethnic groups = 33  Native or Indigenous group = 2  Other ethnic group = 4  White or Caucasian = 682  After exclusion of missing values, used in DIF  Caucasian non-Caucasian  670 [71%] 273 [29%] |
| **ISAAQ severity** | 24.3 (13.4) | 30.25 (12.7) |
| **CIUS** | 8.2 (4.3) | NA |
| **IGDT** | 2.6 (3.8) | 4.47 (4.5) |
| **IAT10** | 17.7 (6.4) | 18.16 (7.1) |

ISAAQ= Internet severity and activities addiction Questionnaire; CIUS=Compulsive Internet Use Scale; IGDT=Internet Gaming Disorder Test; IAT10=Internet Addiction Test, 10-items

## Table S3b - Demographics and descriptive statistics for USA and UK separately

|  | **USA (n=384)** Mean (sd) or [%] | **UK (n=559)** Mean (sd) or count[%] |
| --- | --- | --- |
| **Age** | 23.6 (3.7) | 24.9 (3.5) |
| **Gender** | Male = 191  Female = 193  Non-Binary / Non-specified = 10  After exclusion of non-binary and missing values, used in DIF  193 F 191 M  [50.2%] [ 49.8%] | Male = 208  Female = 351  Non-binary/ Non-specified = 7  After exclusion of non-binary and missing values, used in DIF  351 F 208 M  [62.7%] [37.2%] |
| **Ethnicity** | Asian = 45  Black, African, or Caribbean = 40  Hispanic or Latino = 50  Middle Eastern = 3  Mixed/Multiple ethnic groups = 12  Native or Indigenous group = 2  Other ethnic group = 0  White or Caucasian = 242  After exclusion of missing values, used in DIF  Caucasian non-Caucasian  237 [61.7%] 147 [38.2%] | Asian = 79  Black, African, or Caribbean = 21  Hispanic or Latino = 1  Middle Eastern = 0  Mixed/Multiple ethnic groups = 21  Native or Indigenous group = 0  Other ethnic group = 4  White or Caucasian = 443  After exclusion of missing values, used in DIF  Caucasian non-Caucasian  433 [77.4%] 126 [22.5%] |
| **ISAAQ severity** | 30.67 (12.6) | 29.85 (12.7) |
| **CIUS** | NA | NA |
| **IGDT** | 4.45 (4.6) | 4.45 (4.4) |
| **IAT10** | 18.2 (7.1) | 18.0 (7.1) |

ISAAQ= Internet severity and activities addiction Questionnaire; CIUS=Compulsive Internet Use Scale; IGDT=Internet Gaming Disorder Test; IAT10=Internet Addiction Test, 10-items

## Table S4 - IRT parameters for the Original IRT ISAAQ in SA sample

| **Item** | **Slope** | **b1** | **b2** | **b3** | **b4** | **b5** | **Difficulty** |
| --- | --- | --- | --- | --- | --- | --- | --- |
| **1** | 2.101 | -2.184 | -1.1268 | -0.019 | 0.737 | 1.628 | 0.113 |
| **2** | 1.891 | -1.124 | -0.0841 | 0.804 | 1.489 | 2.33 | 0.842 |
| **3** | 1.908 | -1.958 | -1.012 | -0.035 | 0.749 | 1.654 | 0.151 |
| **4** | 2.635 | -1.207 | -0.081 | 0.790 | 1.418 | 2.292 | 0.904 |
| **5** | 1.175 | 0.134 | 1.618 | 2.596 | 3.326 | 4.307 | 2.22 |
| **6** | 1.193 | 0.735 | 1.8450 | 2.653 | 3.238 | 4.146 | 2.344 |
| **7** | 2.039 | -1.174 | -0.067 | 0.881 | 1.519 | 2.416 | 0.889 |
| **8** | 1.360 | -2.873 | -1.417 | -0.324 | 0.689 | 1.655 | -0.22 |
| **9** | 1.284 | -0.713 | 0.806 | 1.989 | 2.627 | 3.855 | 1.655 |
| **10** | 1.432 | 0.033 | 1.397 | 2.392 | 2.927 | 3.6887 | 1.990 |
| **11** | 2.491 | -1.049 | -0.164 | 0.677 | 1.326 | 2.0675 | 0.826 |
| **12** | 2.324 | -0.371 | 0.6265 | 1.35 | 1.95 | 2.724 | 1.39 |
| **13** | 1.988 | -1.166 | -0.202 | 0.67 | 1.28 | 2.164 | 0.743 |
| **14** | 1.893 | -0.359 | 0.6714 | 1.470 | 2.031 | 3.023 | 1.439 |
| **15** | 1.983 | -1.074 | -0.002 | 0.90 | 1.53 | 2.371 | 0.909 |

## Table S5 - IRT metrics including differential item functioning in the South African sample

|  | **Item drop** | **M2 /**  **RMSEA_2_** | **SRMSR** | **TLI** | **CFI** | **RMSEA.S_X2**  **(item fit)** | **Proportion variance** | **IRT reliability r_xx_ estimate** | **DIF items (age, as binary above or below 25yrs)** | **DIF items (gender, as binary Female or Male)** | **DIF items**  **(ethnicity, as binary Caucasian or non-Caucasian)** |
| --- | --- | --- | --- | --- | --- | --- | --- | --- | --- | --- | --- |
| **ISAAQ**  **i15** | Null | 1980.3 /0.08 | 0.049 | 0.97 | 0.97 | <0.05 all items | 0.524 | 0.93 | 1,4,**5**,**6**,7, **9**,13,15 | 2,**5**,**6,7,8**, 10,11,12 | 1,2,**6, 8,9**,12 |
| **ISAAQ**  **i14** | 5 | 1720.7 /0.08 | 0.046 | 0.97 | 0.97 | <0.05 all items | 0.538 | 0.93 | 1,3,4,**6**,  7,**9**,13 | 2,**6**,**10**, 11,12,13 | 1,2,**6**,**8,9,10**,12 |
| **ISAAQ**  **i13** | 5,6 | 1545.8 /0.08 | 0.043 | 0.97 | 0.98 | <0.05 all items | 0.554 | 0.93 | 1,3,7,  **9**,13 | 2,7,**10**, 11,12,13 | 1,2,**8**,**9**,12 |
| **ISAAQ**  **i12** | 5, 6, 9 | 1309.7 /0.08 | 0.039 | 0.97 | 0.98 | <0.05 all items | 0.571 | 0.93 | 1,3,7,13 | 2,7,**10**,11  ,12,13 | 1,2,**8**,**10**,12,13 |
| **ISAAQ**  **i11** | 5, 6, 8, 9 | 1287.9 /0.09 | 0.042 | 0.97 | 0.98 | <0.05 all items | 0.587 | 0.925 | 1,3,7,13 | 2,**10**,11,12,13 | 1,2,**10**,12 |
| **ISAAQ**  **i10** | 5, 6, 8, 9, 10 | 1145.7 /0.09 | 0.043 | 0.97 | 0.97 | <0.05 all items | 0.608 | 0.923 | 1,3,7,13 | 2,7,11,12,13 | 1,2,12 |
| **ISAAQ**  **i9** | 5, 6, 8, 9, 10, 2 | 738.1 /0.09 | 0.036 | 0.97 | 0.98 | <0.05 all items | 0.613 | 0.917 | 1,3,7,13,15 | 7, 11, 12, 13 | 1, 12 |
| **ISAAQ**  **i8** | 5, 6, 8, 9, 10, 2, 1 | 613.4 /0.09 | 0.038 | 0.97 | 0.98 | <0.05 all items | 0.614 | 0.906 | 3, 4, 7, 13 | 3, 12, 14, 15 | 12, 13 |

ISAAQ= Internet severity and activities addiction Questionnaire; IRT = Item Response Theory; RMSEA=Root-Mean-squared-Error Approximation; CFI = Comparative Fit Index; TLI = Tucker-Lewis Index; SRMR = Standardized Root Mean Squared Error; DIF=Differential Item Functioning

## Table S6 - IRT metrics including differential item functioning in the USA-UK sample

|  | **Item drop** | **M2 /**  **RMSEA_2_** | **SRMSR** | **TLI** | **CFI** | **RMSEA.S_X2**  **(item fit)** | **Proportion variance** | **IRT reliability r_xx_ estimate** | **DIF items (age, as binary above or below 25yrs)** | **DIF items (gender, as binary Female or Male)** | **DIF items**  **(ethnicity, as binary Caucasian or non-Caucasian)** |
| --- | --- | --- | --- | --- | --- | --- | --- | --- | --- | --- | --- |
| **ISAAQ**  **i15** | Null | 915.2 /0.09 | 0.061 | 0.94 | 0.95 | <0.05 all items | 0.46 | 0.91 | 1, 2, **5**, 13 | 1,2,**6,8,10,**12,15 | **8,10** |
| **ISAAQ**  **i14** | 5 | 851.6 /0.09 | 0.063 | 0.94 | 0.95 | <0.05 all items | 0.47 | 0.916 | 1, 2, 13 | 1,2,**6**,**8,10**,12,15 | **8, 10** |
| **ISAAQ**  **i13** | 5,6 | 773.9 /0.10 | 0.064 | 0.94 | 0.94 | <0.05 all items | 0.48 | 0.913 | 1, 2, 13 | 2,3,**8,10**,12,15 | **10** |
| **ISAAQ**  **i12** | 5, 6, 9 | 699.9 /0.10 | 0.065 | 0.93 | 0.94 | <0.05 all items | 0.49 | 0.901 | 1, 13 | 1,2**,8,10,** 12,14,15 | **10** |
| **ISAAQ**  **i11** | 5, 6, 8, 9 | 622.6 /0.11 | 0.066 | 0.93 | 0.94 | <0.05 all items | 0.50 | 0.905 | 1, 13 | 1,2,**10**,12,15 | **10** |
| **ISAAQ**  **i10** | 5, 6, 8, 9, 10 | 556.8 /0.11 | 0.068 | 0.92 | 0.94 | <0.05 all items | 0.51 | 0.899 | 1, 13 | 2, 12, 15 | null |
| **ISAAQ**  **i9** | 5, 6, 8, 9, 10, 2 | 343.9 /0.11 | 0.591 | 0.93 | 0.95 | <0.05 all items | 0.51 | 0.891 | 13 | 12, 15 | null |
| **ISAAQ**  **i8** | 5, 6, 8, 9, 10, 2, 1 | 219.8 /0.10 | 0.51 | 0.95 | 0.96 | <0.05 all items | 0.52 | 0.886 | 13 | 12, 15 | null |

ISAAQ= Internet severity and activities addiction Questionnaire; IRT = Item Response Theory; RMSEA=Root-Mean-squared-Error Approximation; CFI = Comparative Fit Index; TLI = Tucker-Lewis Index; SRMR = Standardized Root Mean Squared Error; DIF=Differential Item Functioning

### Figure S1a – DIF examination: age


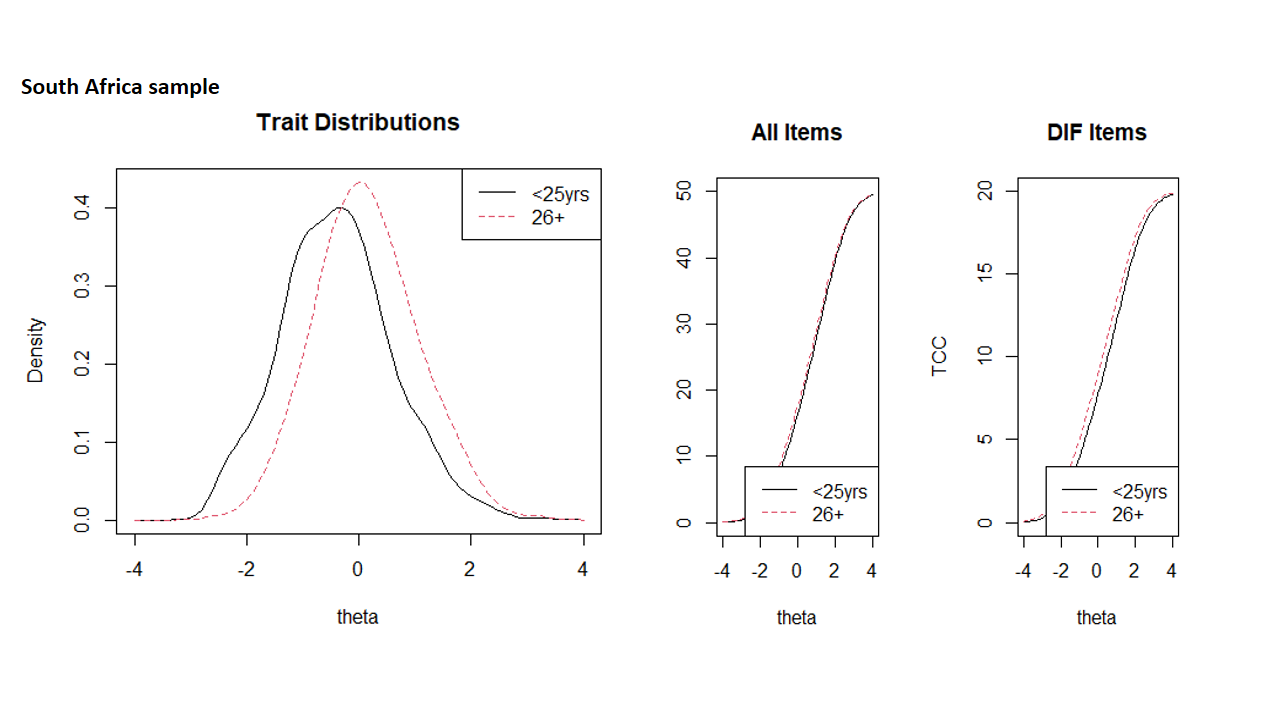

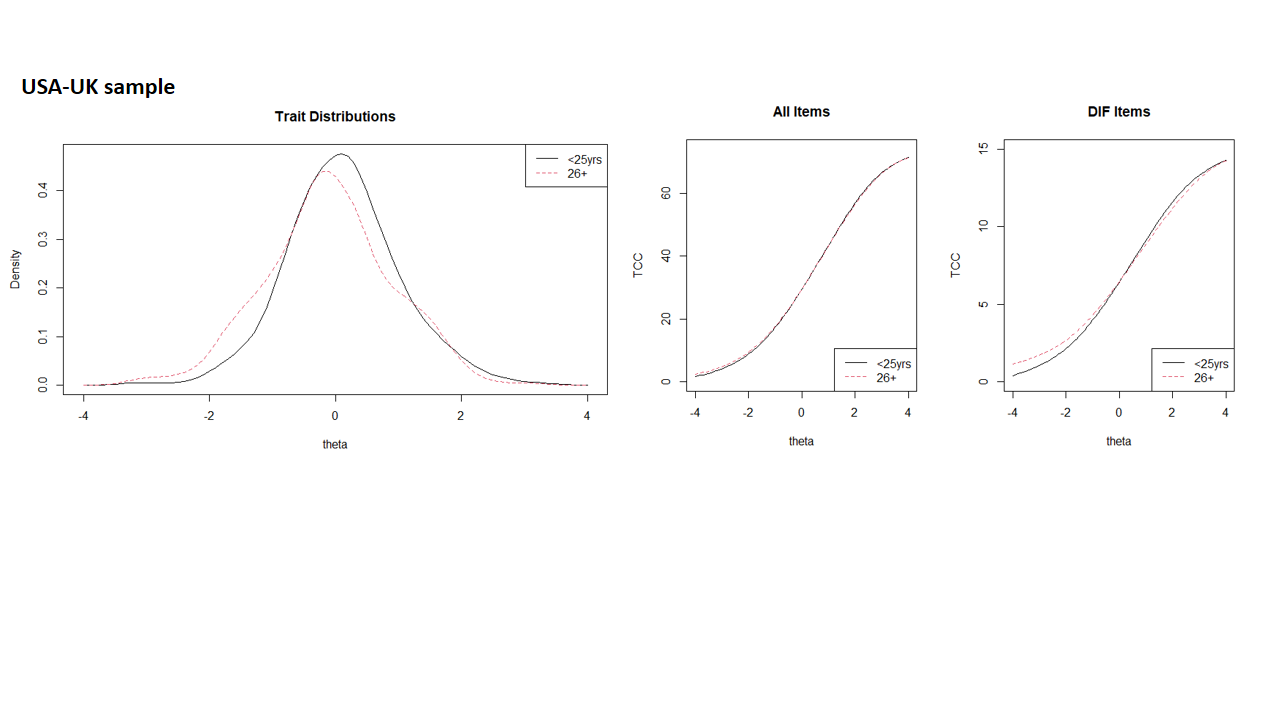


### Figure S1b – DIF examination: gender

**
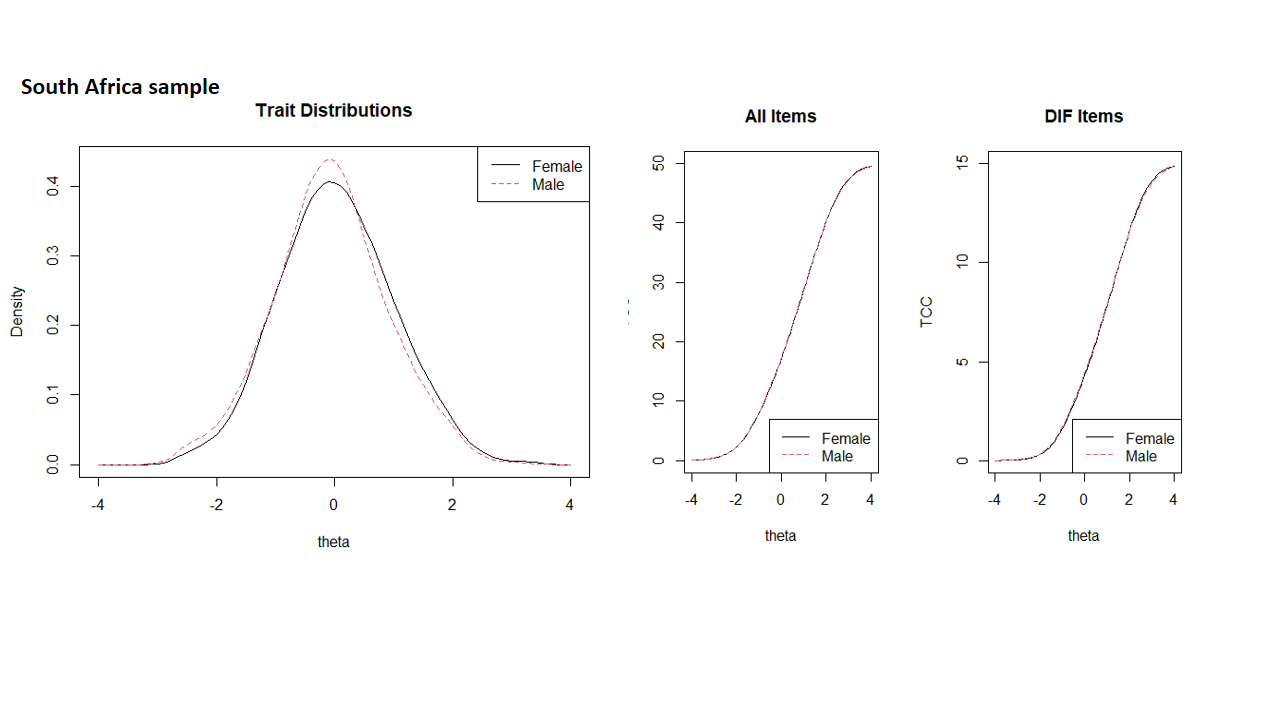

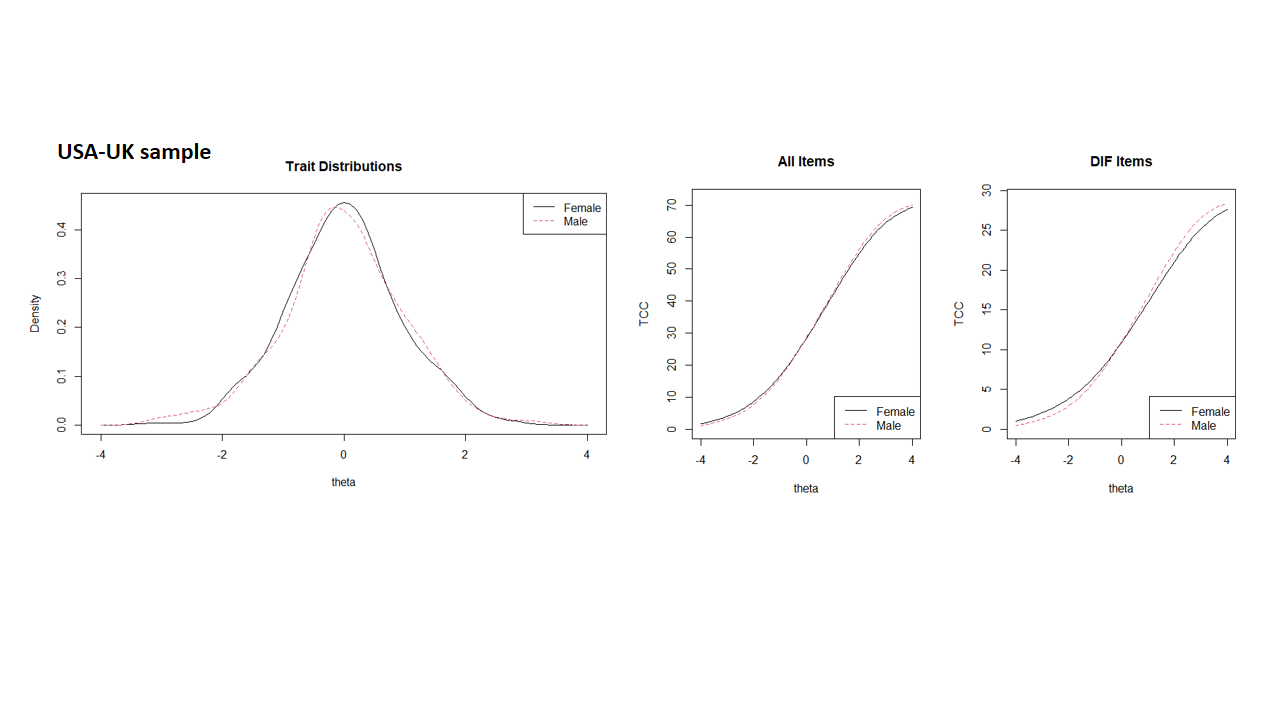
**

### Figure S1c – DIF examination: race


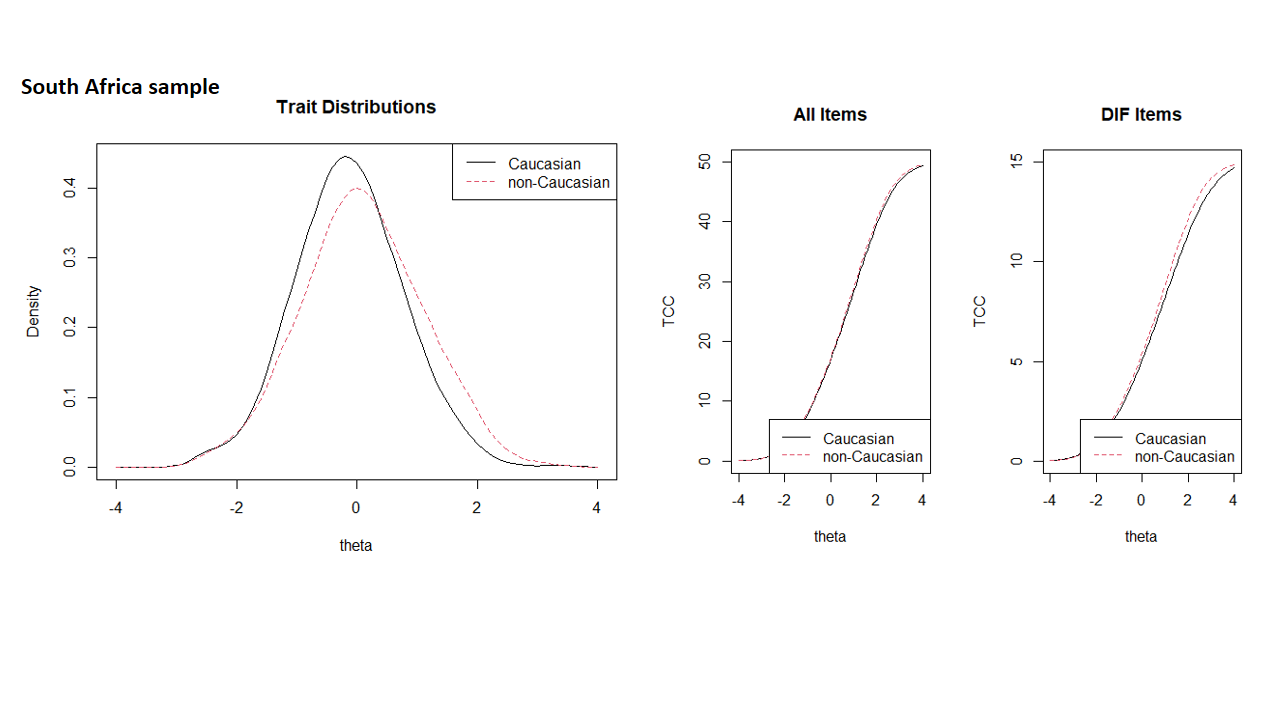

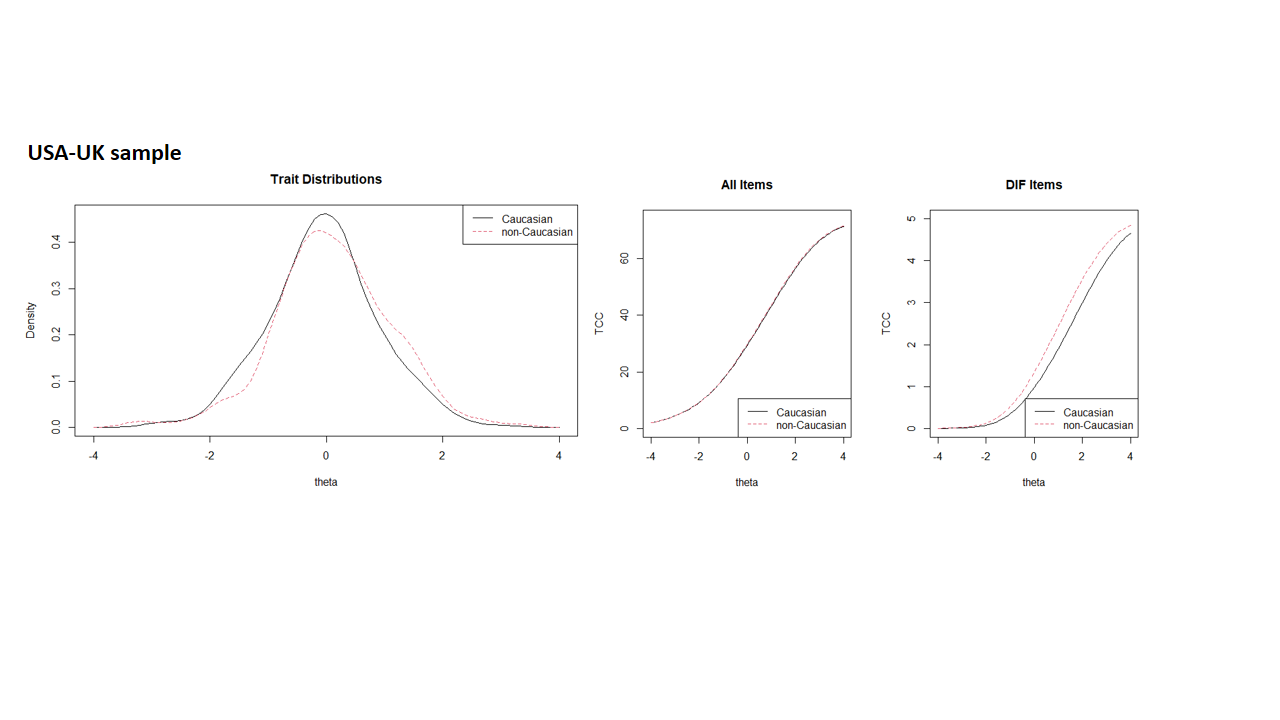


## Figure S2- Scree plot for original ISAAQ and ISAAQ-10 exploratory factor analysis


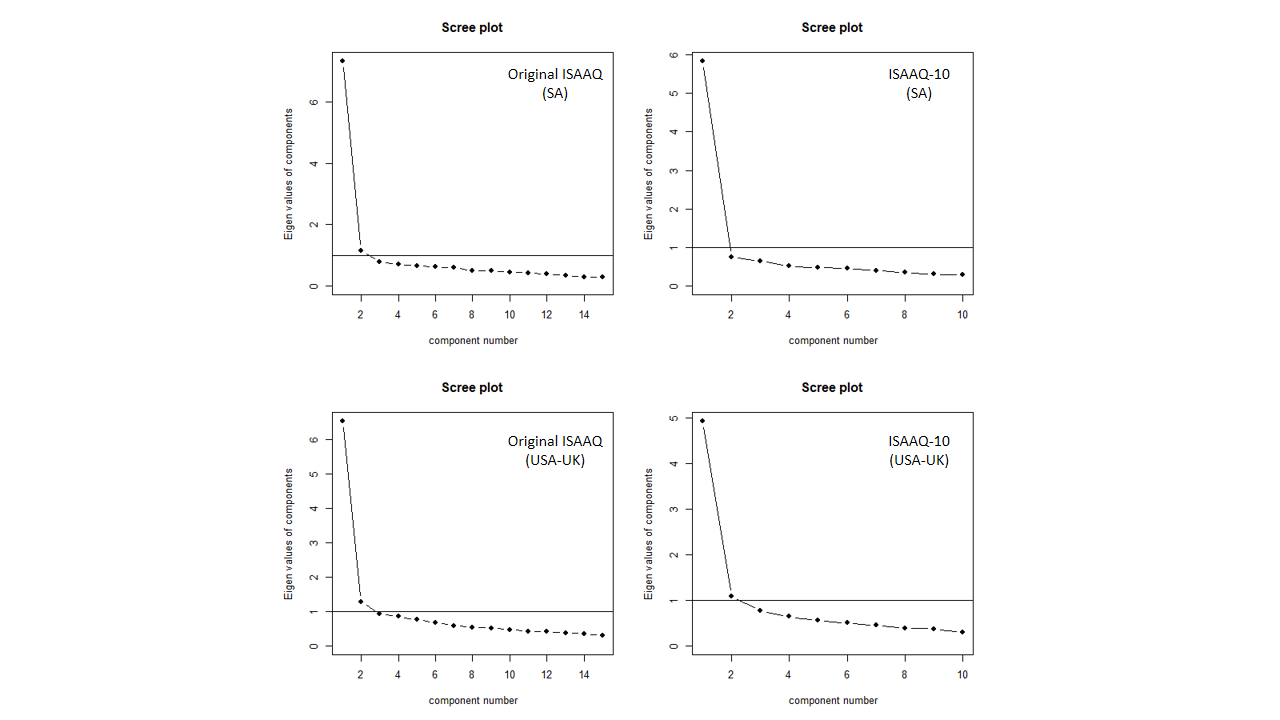


## Table S7 - Clustering analysis in the South Africa sample

| *cluster* | *SURF* | *GAME* | *SGTW* | *SHOP* | *GAMBL* | *SNS* | *CYCHR* | *PORN* | *STREM* | *CYBUL* | *AGE* | *gender* | *ISAAQ* | *IAT10* | *IGDT* | *#* |
| --- | --- | --- | --- | --- | --- | --- | --- | --- | --- | --- | --- | --- | --- | --- | --- | --- |
| 1 | -0.01 | -0.02 | -0.02 | -0.01 | -0.04 | 0 | -0.01 | -0.03 | 0 | -0.11 | 24.28 | 1.65 | 23.97 | 17.45 | 2.55 | 3213 |
| 2 | 0.34 | 0.98 | 0.83 | 0.61 | 2.18 | 0.17 | 0.51 | 1.4 | 0.25 | 5.95 | 22.37 | 1.44 | 38.15 | 25.11 | 7.27 | 62 |

SURF = General surfing; GAME = Online gaming; SGTW = Skill games and time wasters online; SHOP = Online shopping; GAMBL = Online gambling; SNS = Online social media use; CYCHR = Cyberchondria; PORN = Online pornography use; STREM = Online Streaming; CYBUL = Cyberbullying (perpetration); AGE = participant age; ISAAQ = ISAAQ-15 scores; IAT10 = Internet Addiction Test 10-item score; IGDT = Internet Gaming Disorder Test score.

## Table S8 - Clustering analysis in the UK/USA sample

| **CLUSTER** | **SURF** | **GAME** | **SGTW** | **SHOP** | **GAMBL** | **SNS** | **CYCHR** | **PORN** | **STREM** | **CYBUL** | **AGE** | **GENDER** | **ISAAQ** | **IAT10** | **IGDT** | **#** |
| --- | --- | --- | --- | --- | --- | --- | --- | --- | --- | --- | --- | --- | --- | --- | --- | --- |
| **1** | 0 | -0.02 | -0.01 | -0.01 | -0.04 | 0 | -0.02 | -0.01 | -0.01 | -0.1 | 24.43 | 0.42 | 29.95 | 17.92 | 4.36 | 928 |
| **2** | 0.04 | 0.96 | 0.86 | 0.82 | 2.35 | 0.02 | 1.31 | 0.66 | 0.52 | 6.04 | 25.27 | 0.87 | 44.73 | 29.6 | 10.47 | 15 |

### Figure S3- Elbow plot and silhouette plot in the SA sample


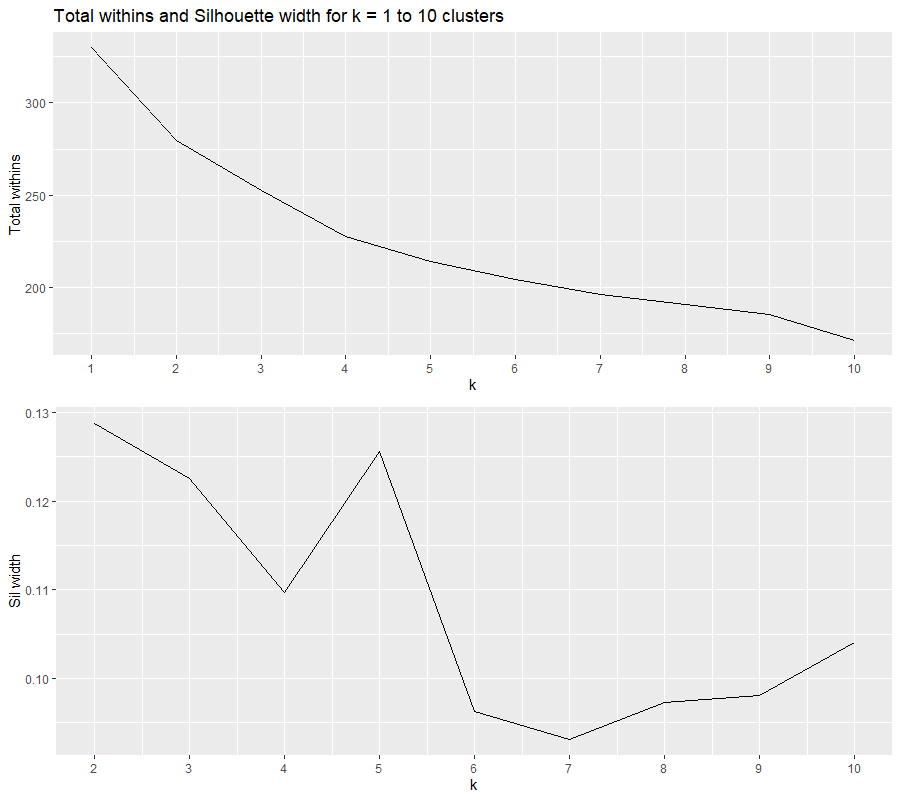


### Figure S4 - Elbow plot and silhouette plot in the USA-UK sample


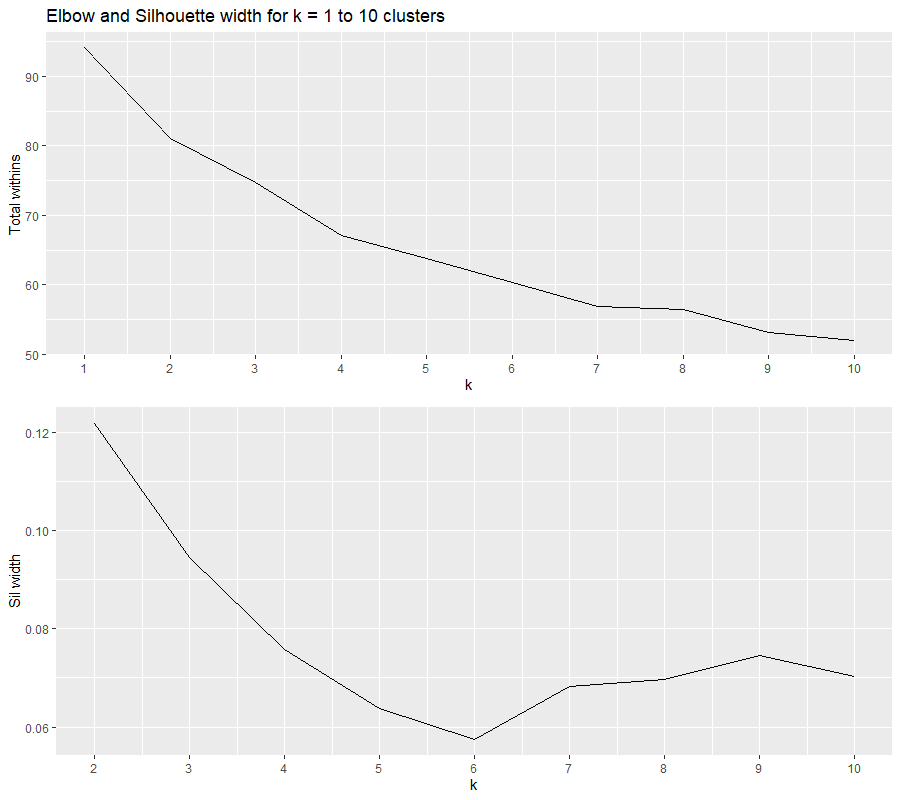


## §S1 - Determining the number of clusters by majority vote using Nbclust

* ***South Africa sample***: Among all 26 clustering indices (KL, CH, Hartigan, CCC, Scott, Marriot, TrCovW, TraceW, Friedman, Rubin, Cindex, DB, Silhouette, Duda, PseudoT2, Beale, Ratkowsky, Ball, Pt, Biserial, Frey, McClain, Dunn, Hubert, SDindex, Dindex, SDbw)

*** 6 proposed 2 as the best number of clusters**

* 6 proposed 3 as the best number of clusters

* 5 proposed 4 as the best number of clusters 🡪 According to the majority rule, **the best number of clusters is 2**

* 4 proposed 5 as the best number of clusters

* 2 proposed 9 as the best number of clusters

* 1 proposed 10 as the best number of clusters

***USA-UK sample*** Among all indices:

* **11 proposed 2 as the best number of clusters**

* 8 proposed 3 as the best number of clusters

* 3 proposed 4 as the best number of clusters 🡪 According to the majority rule, **the best number of clusters is 2**

* 1 proposed 6 as the best number of clusters

* 1 proposed 10 as the best number of clusters

### Figure S5 - Three clusters, alternative


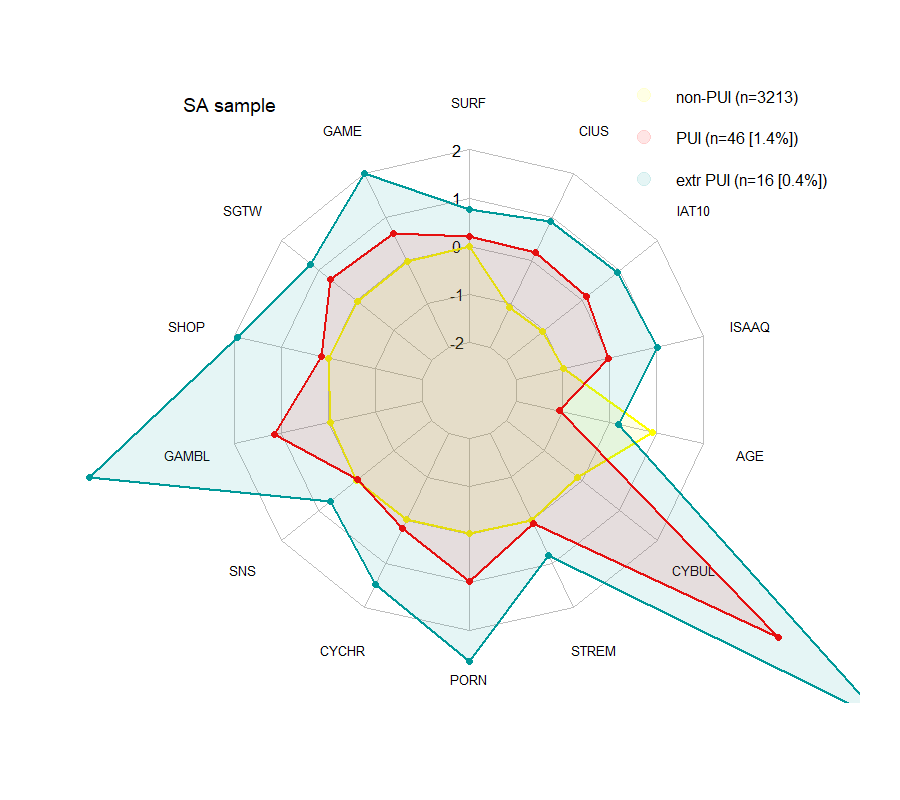

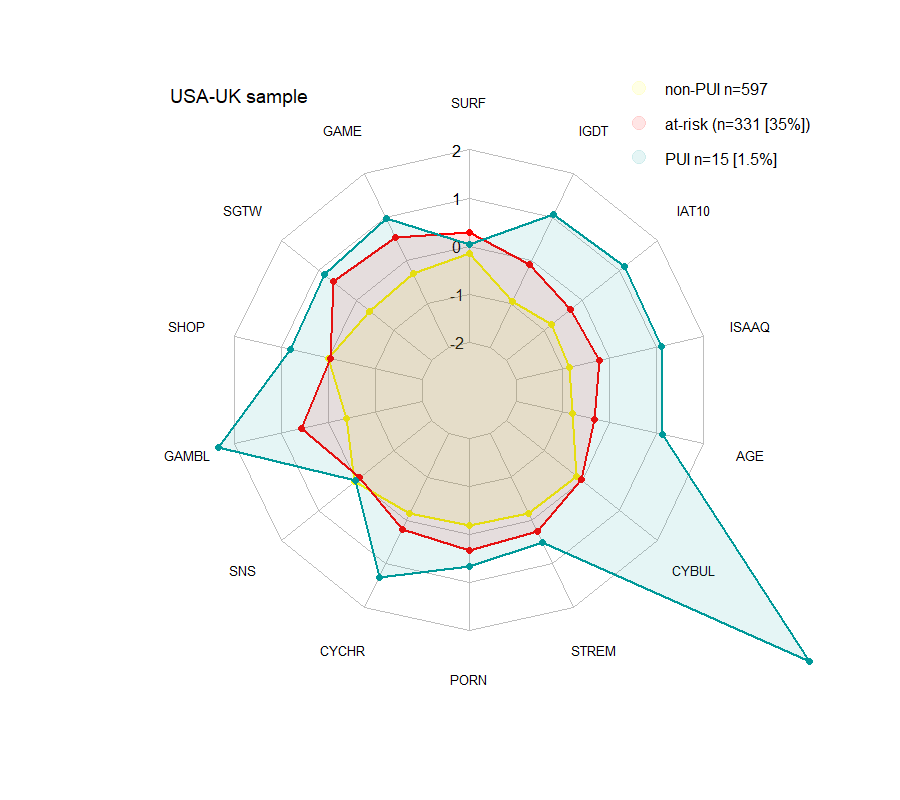


Those plots were made to explore whether the PUI cluster was robust to choosing three clusters (second best option). To note that the SA sample has on average, much lower scores of problematic usage of the internet compared to the USA-UK sample (mean(sd) 24.3 (13.4) vs. 30.25 (12.7)). SURF = General surfing; GAME = Online gaming; SGTW = Skill games and time wasters online; SHOP = Online shopping; GAMBL = Online gambling; SNS = Online social media use; CYCHR = Cyberchondria; PORN = Online pornography use; STREM = Online Streaming; CYBUL = Cyberbullying (perpetration); AGE = participant age; ISAAQ = ISAAQ-15 scores; IAT10 = Internet Addiction Test 10-item score; IGDT = Internet Gaming Disorder Test score.

## Extra materials

## §S2 Extreme responding examination

In this analysis we used the Short Version of the Problematic Mobile Phone Use Questionnaire (PMPUQ-SV).
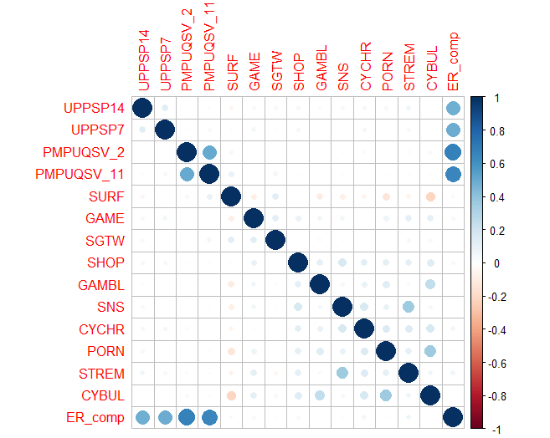
Extreme responding is a common issue in survey questionnaires. To explore that in this dataset and particularly to examine extreme responding in the ISAAQ Part B (activities component) we used an applied variation of the method proposed by Greenleaf (1992). In this simple approach we carefully select items from different instruments of our survey (with correlation close to zero with the ISAAQ Part B items) to form a composite score of ER. We did not have any measure in 6-point Likert suitable for this use, therefore the best suitable options in that regard were 4-likert point items from S-UPPS and PMPUQ-SV; SUPPS item 14, SUPPS item7, PMPUQ-SV item 2 and PMPUQ-SV item 11. Original scores are recoded so that they reflect counts of extreme responses, with them then aggregated to produce an extreme response metric. For example, if the original Likert scale is (1-4) it is then transformed to 1, 0, 0, 1 or if it is (0-5) then it is transformed into 1, 0, 0, 0, 0, 1, counting only extreme responses as "1". Correlations of this ER is then checked against the ISAAQ Part B items. None of the ISAAQ B items correlated with the ER. All items |r| <0.06. See figure S4.

## Figure S6 – ICC in the USA-UK sample


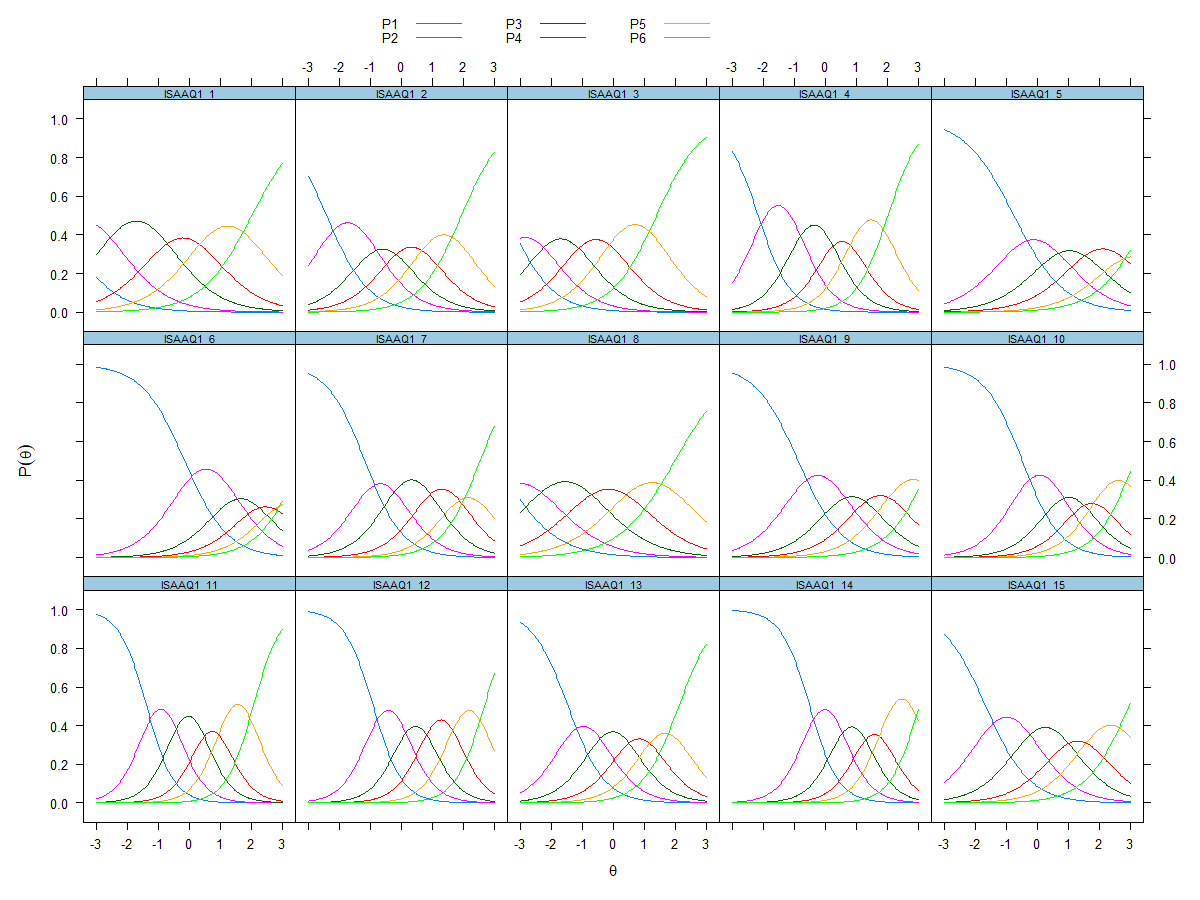


## Figure S7 – IIC in the USA-UK sample


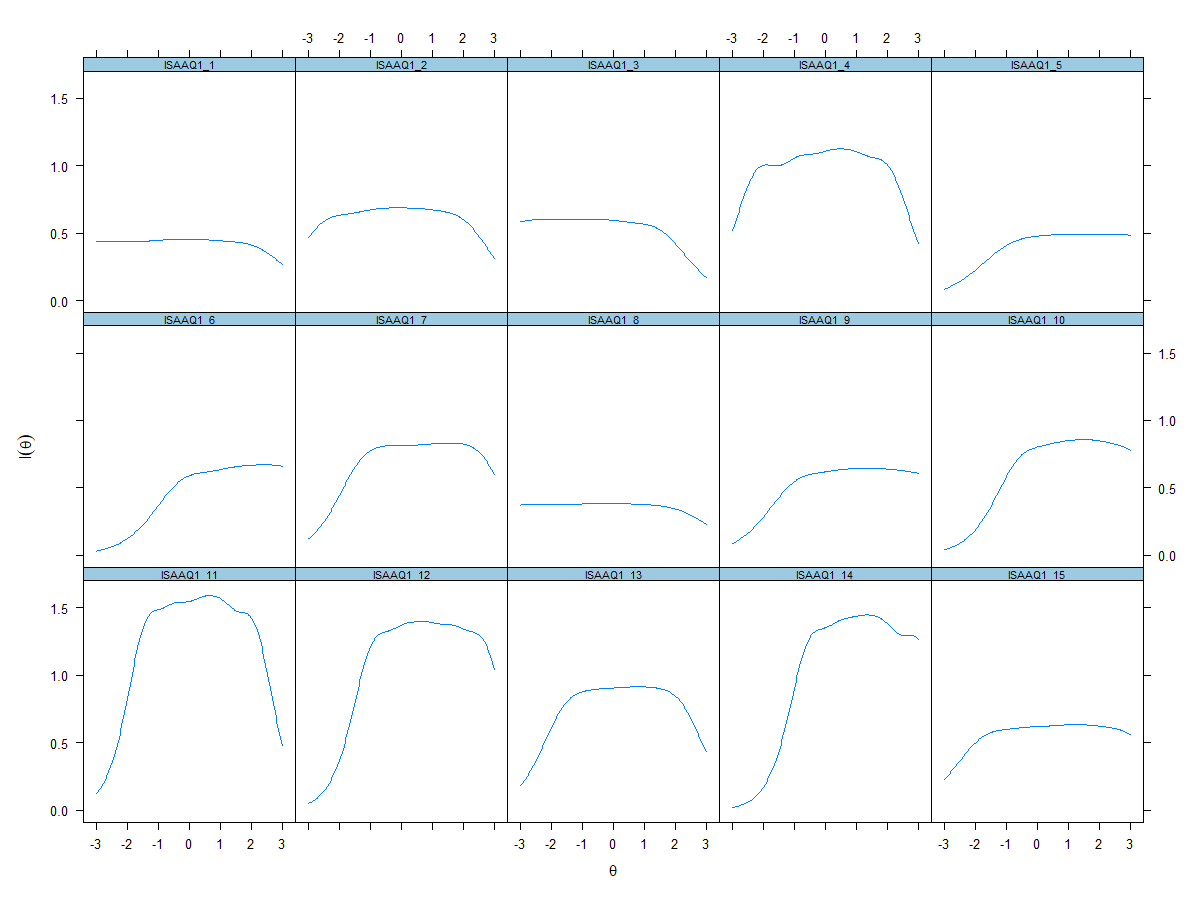


## Figure S8 – Raincloudplots for main variables and age for each site
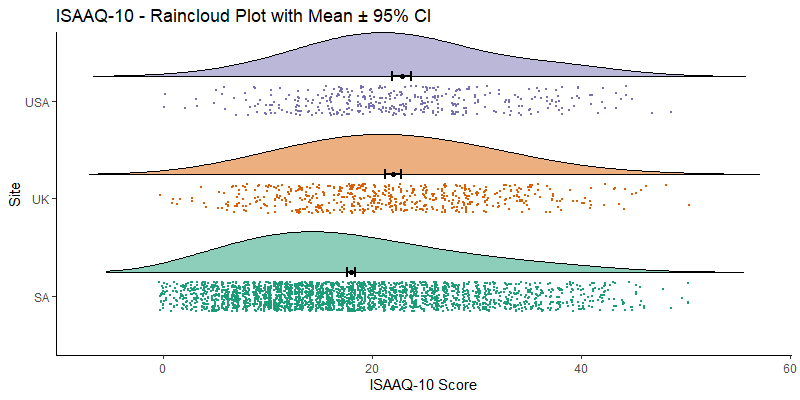

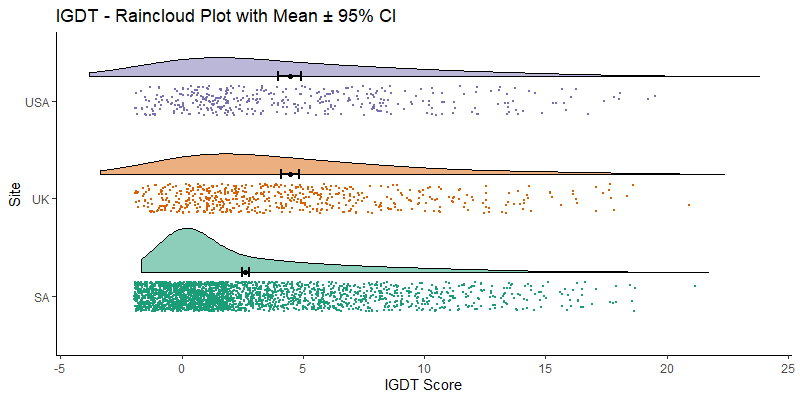

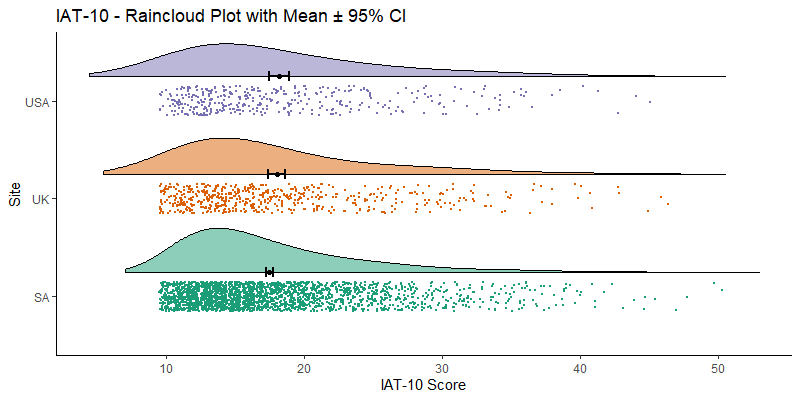

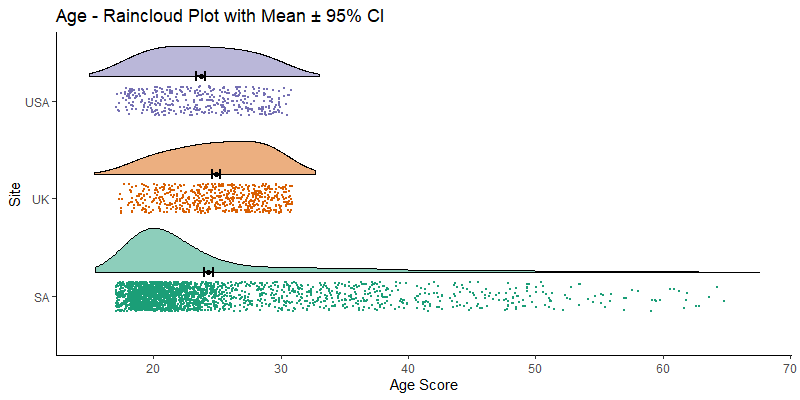


## Figure S9 – Differential test functioning between USA and UK sites


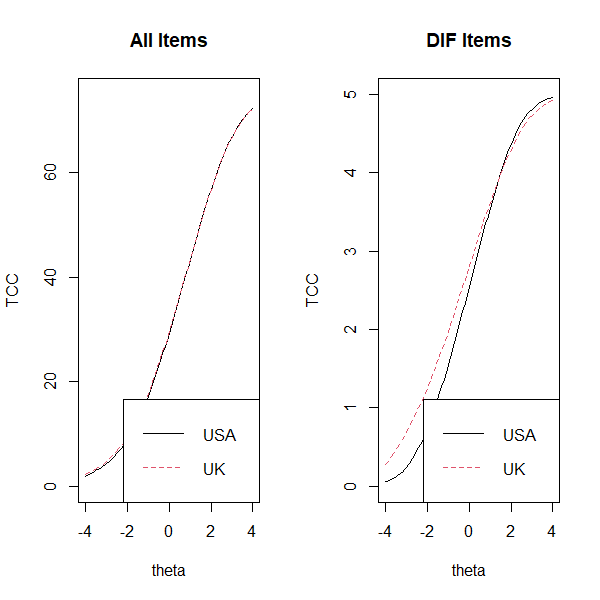

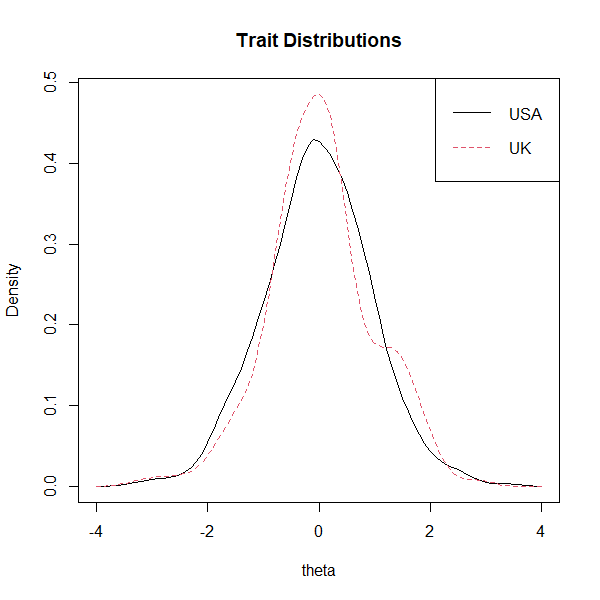

Supplement: Supplementary file 1 — Supplementary material [file mmc1.docx]
